# Supplementary material for: Goal-directed navigation in humans and deep reinforcement learning agents relies on an adaptive mix of vector-based and transition-based strategies
Source: PLoS Biol. 2025 Jul 29;23(7):e3003296. doi: 10.1371/journal.pbio.3003296 (PMC12324678; doi:10.1371/journal.pbio.3003296)
Supplement: S1 Fig — B: Schematic diagram of the map reading phase in the free sampling condition in Day 2 of Experiment 3. C: Schematic diagram for the navigation phase. D: View when the directions display was disabled in Experiment 1. E: View when the states display was disabled in Experiment 1. F: Schematic diagram for the memory probe phase in Experiment 3. (PDF) [file pbio.3003296.s001.pdf]

Supplementary Figure 1: Task Schematics

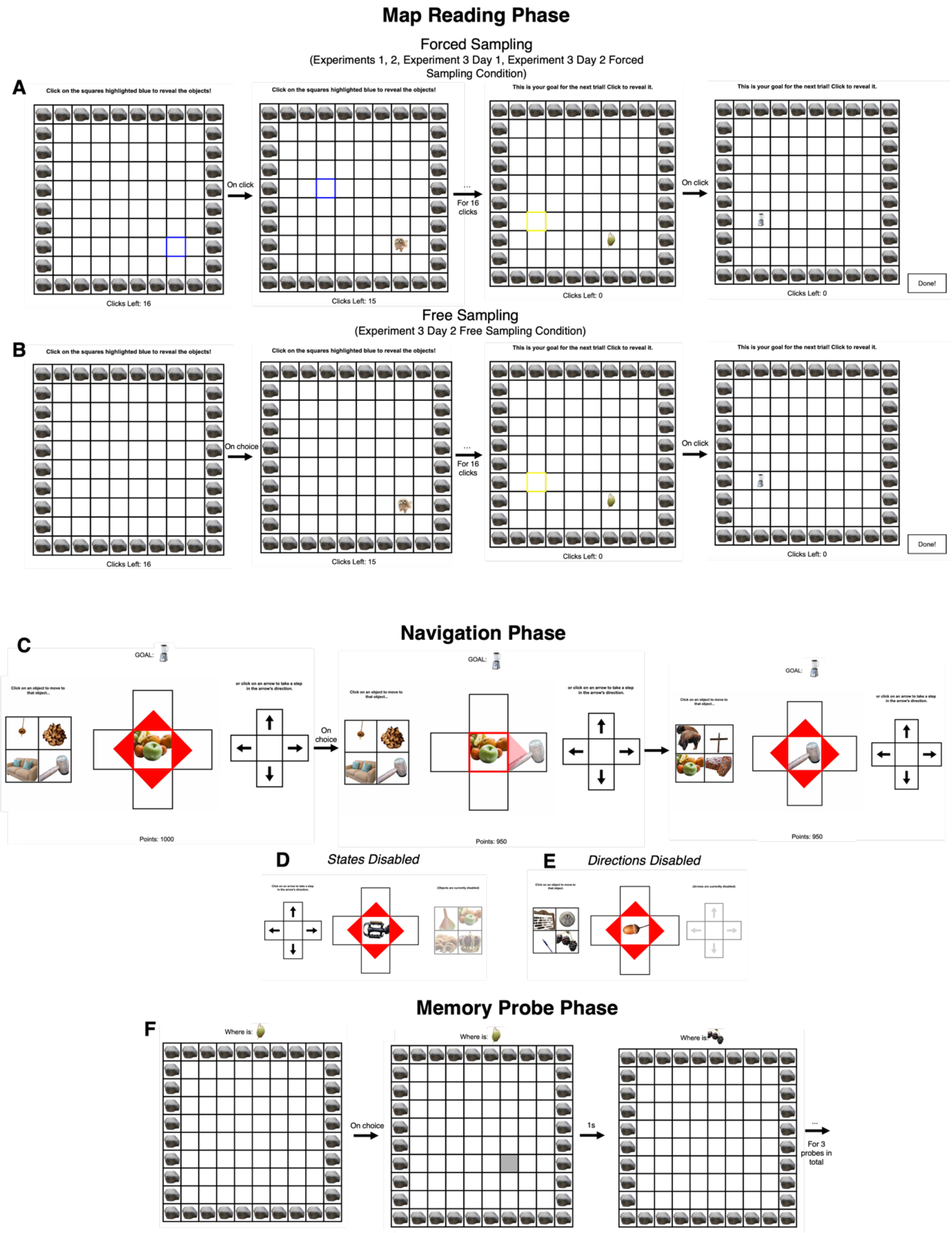


*Figure S1:* A: Schematic diagram of the map reading phase in Experiments 1 and 2, Day 1 of Experiment 3, and the forced sampling condition in Day 2 of Experiment 3. B: Schematic diagram of the map reading phase in the free sampling condition in Day 2 of Experiment 3. C: Schematic diagram

for the navigation phase. D: View when the *directions* display was disabled in Experiment 1. E: View when the *states* display was disabled in Experiment 1. F: Schematic diagram for the memory probe phase in Experiment 3.
