# Supplementary material for: Goal-directed navigation in humans and deep reinforcement learning agents relies on an adaptive mix of vector-based and transition-based strategies
Source: PLoS Biol. 2025 Jul 29;23(7):e3003296. doi: 10.1371/journal.pbio.3003296 (PMC12324678; doi:10.1371/journal.pbio.3003296)
Supplement: S2 Fig — Data underlying this figure is available at https://osf.io/w39d5/. (PDF) [file pbio.3003296.s002.pdf]

Supplementary Figure 2: Response Type by Environment Type

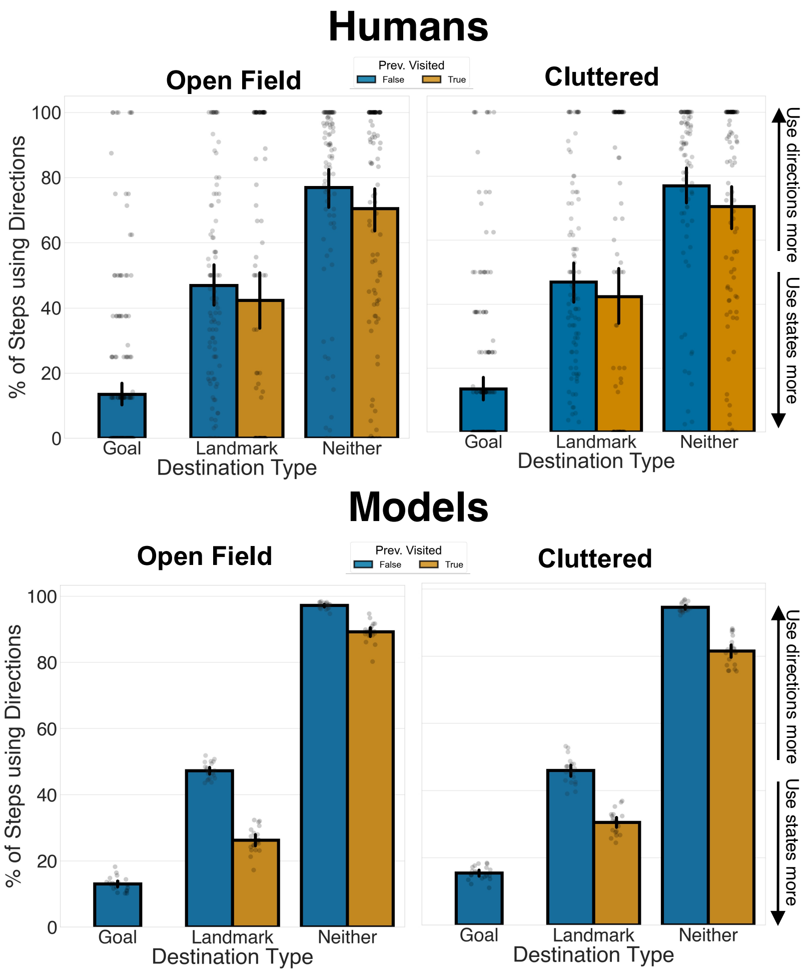


*Figure S2:* Participants' (above) and models' (below) use of vector-based responses (y-axis) as a function of destination type (i.e., goal, landmark, or non-landmark; x-axis) and whether the state had been visited before (colour of bar), split by whether navigation was in an open field (left) or cluttered (right) environment. Data underlying this figure is available at https://osf.io/w39d5/.
