# Supplementary material for: Goal-directed navigation in humans and deep reinforcement learning agents relies on an adaptive mix of vector-based and transition-based strategies
Source: PLoS Biol. 2025 Jul 29;23(7):e3003296. doi: 10.1371/journal.pbio.3003296 (PMC12324678; doi:10.1371/journal.pbio.3003296)
Supplement: S3 Fig — Each point represents a single person, while the lines represent the best-fitting quadratic curve. C/D: Participants’ use of vector-based responses (y-axis) as a function of destination type (i.e., goal, landmark, or non-landmark; x-axis) and whether the state had been visited before (color of bar) in Experiment 2 (C) and Experiment 3 (D). Data underlying this figure is available at https://osf.io/w39d5/. (PDF) [file pbio.3003296.s003.pdf]

Supplementary Figure 3: Replications and Pre-registered Results

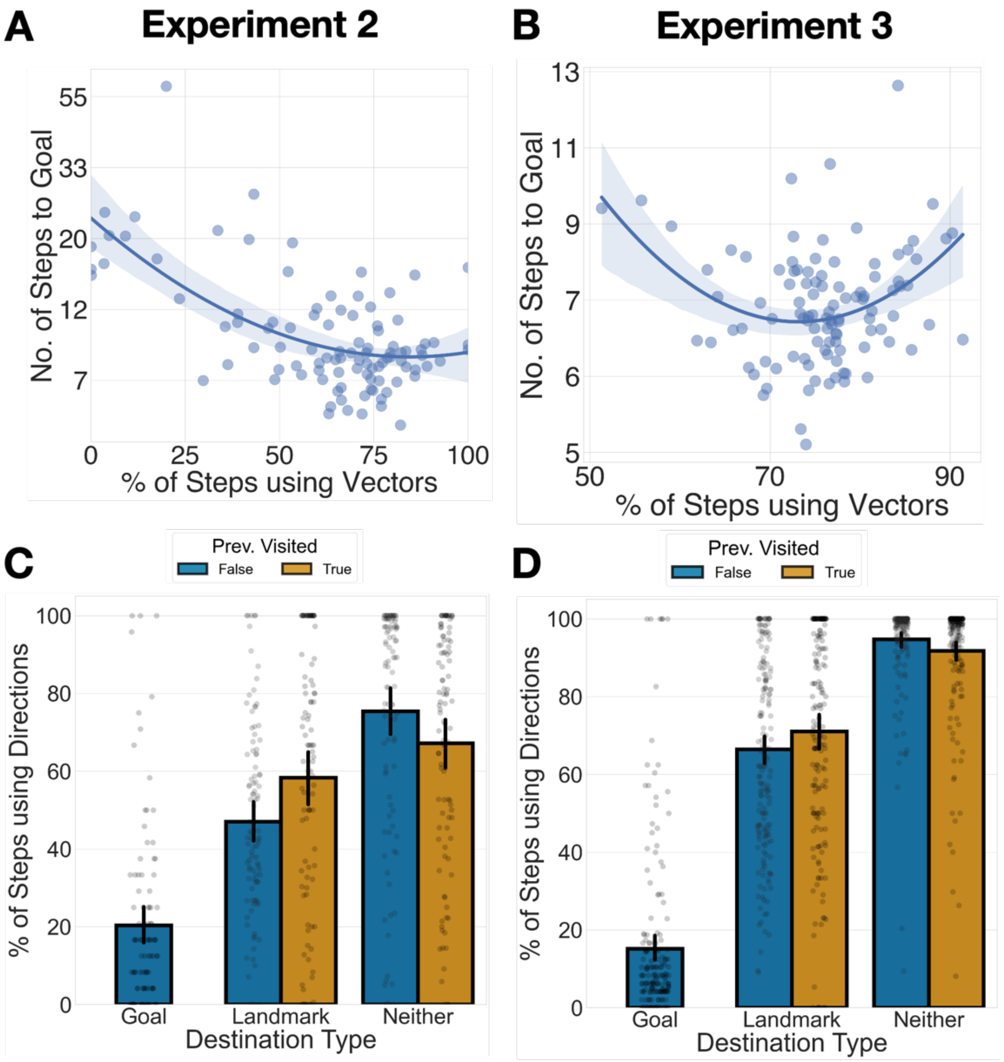


*Figure S3:* A/B: Relationship between the proportion of steps made using vector-based responses (x-axis) and the number of steps taken to reach a goal (y-axis; represented on a logarithmic scale) for each participant in Experiment 2 (A) and Experiment 3 (B). Each point represents a single person, while the lines represent the best-fitting quadratic curve. C/D: Participants' use of vector-based responses (y-axis) as a function of destination type (i.e., goal, landmark, or non-landmark; x-axis) and whether the state had been visited before (colour of bar) in Experiment 2 (C) and Experiment 3 (D). Data underlying this figure is available at https://osf.io/w39d5/.

## Quadratic Relationship between Use of Vectors and Performance

Experiment 2:

A linear regression between overall log-transformed steps to goal across the entire task and linear and quadratic terms for proportion of steps made using vector-based responses replicated the significant association between performance and both the linear term ($\beta$ = -0.62, *SE* = 0.18, $t(97)$ = -3.53, $p$ = .001) and the quadratic term ($\beta$ = 1.37, *SE* = 0.45, $t(97)$ = 3.03, $p$ = .003).

Our pre-registration document indicated that we would use a linear mixed effects model instead. This led to a similar conclusion: there were significant associations between log-transformed number of steps and the linear term for proportion of steps made using vector-based responses ($\beta$ = -0.39, *SE* = 0.16, $z$ = -2.44, $p$ = .01) and the quadratic term ($\beta$ = 1.23, *SE* = 0.36, $z$ = 3.42, $p$ = .0006). Similar results are obtained for a mixed effects logistic regression predicting accuracy (i.e., whether a step is made in the correct direction) for the linear term ($\beta$ = -0.28, *SE* = 0.11, $z$ = -2.40, $p$ = .02) and the quadratic term ($\beta$ = 0.69, *SE* = 0.28, $z$ = 2.50, $p$ = .01). Note that although the pre-registration document mentions using a 'linear mixed effects model' for accuracy, this should have been a mixed effects logistic regression model instead.

Experiment 3:

A linear regression between overall log-transformed steps to goal across the entire task and linear and quadratic terms for proportion of steps made using vector-based responses replicated the significant association between performance and both the linear term ($\beta$ = 0.55, *SE* = 0.16, $t(182)$ = 3.47, $p$ = .001) and the quadratic term ($\beta$ = 3.16, *SE* = 0.44, $t(182)$ = 7.18, $p < .001$).

**Use of Transition-Based Strategies at Landmarks and Goals**

Experiment 2:

We had pre-registered the hypothesis that participants would use transition-based responses more at landmarks and goals. This was confirmed by our pre-registered mixed effects logistic regression model using trial number and destination state type (goal, landmark, or non-landmark) as predictors of response type (transition-based or vector-based). The model suggested that participants were more likely to use a transition-based response at goals ($\beta = 3.96$, $SE = 0.29$, $z = 13.54$, $p < .0001$) and at landmarks ($\beta = 1.63$, $SE = 0.18$, $z = 8.90$, $p < .0001$).

Moreover, we also performed the additional analyses we reported in the main text for Experiment 1 and replicated all main analyses. A mixed effects logistic regression again found that participants were more likely to use state-based responses at goals ($\beta = 3.88$, $SE = 0.31$, $z = 12.55$, $p < .0001$) and landmarks, ($\beta =1.82$, $SE = 0.18$, $z = 10.19$, $p < .0001$). The model also replicated the results that participants were more likely to use state-based responses to get to a state they had previously visited ($\beta = 0.50$, $SE = 0.10$, $z = 4.74$, $p < .0001$), and that this association was attenuated when the destination state was a learnt landmark (interaction term: $\beta = -1.02$, $SE = 0.13$, $z = -7.99$, $p < .0001$).

Experiment 3:

A mixed effects logistic regression replicated the effects we reported in Experiment 1. Participants were more likely to use a state-based response at goals ($\beta = 7.07$, $SE = 0.21$, $z = 33.17$, $p < .0001$) and landmarks ($\beta = 3.33$, $SE = 0.17$, $z = 19.07$, $p < .0001$).

Participants use state-based responses at previously encountered states ($\beta$ = 0.58, *SE* = 0.08, $z$ = 7.70, $p$ < .0001), but this effect was reversed when the state was a landmark ($\beta$ = -0.89, *SE* = 0.10, $z$ = -8.59, $p$ < .0001).

**Individual Difference Measures**

In our pre-registration, we hypothesised that there would be a correlation between participants' Santa Barbara Sense of Direction (SBSOD) scores and their tendency to use vector-based responses, and that this would lead to a similar quadratic relationship between SBSOD scores and performance on the task. However, this did not turn out to be supported by our data. A linear regression suggested that there was no significant association between SBSOD scores and the proportion of steps that participants made using a vector-based response across the experiment ($\beta$ = 0.21, *SE* = 0.47, $t(98)$ = 0.44, $p$ = .66). In the mixed effects linear model analysis that we had pre-registered, there were no significant effects of either the linear term for SBSOD scores responses ($\beta$ = -0.02, *SE* = 0.-3, $z$ = -0.70, $p$ = .48) or the quadratic term ($\beta$ = 0.018, *SE* = 0.018, $z$ = 1.00, $p$ = .32) on log-transformed number of steps.
