# Supplementary material for: Goal-directed navigation in humans and deep reinforcement learning agents relies on an adaptive mix of vector-based and transition-based strategies
Source: PLoS Biol. 2025 Jul 29;23(7):e3003296. doi: 10.1371/journal.pbio.3003296 (PMC12324678; doi:10.1371/journal.pbio.3003296)
Supplement: S4 Fig — Each dot represents and individual participant and the line represents the best-fitting line from a linear regression. Data underlying this figure is available at https://osf.io/w39d5/. (PDF) [file pbio.3003296.s004.pdf]

Supplementary Figure 4: Individual Differences

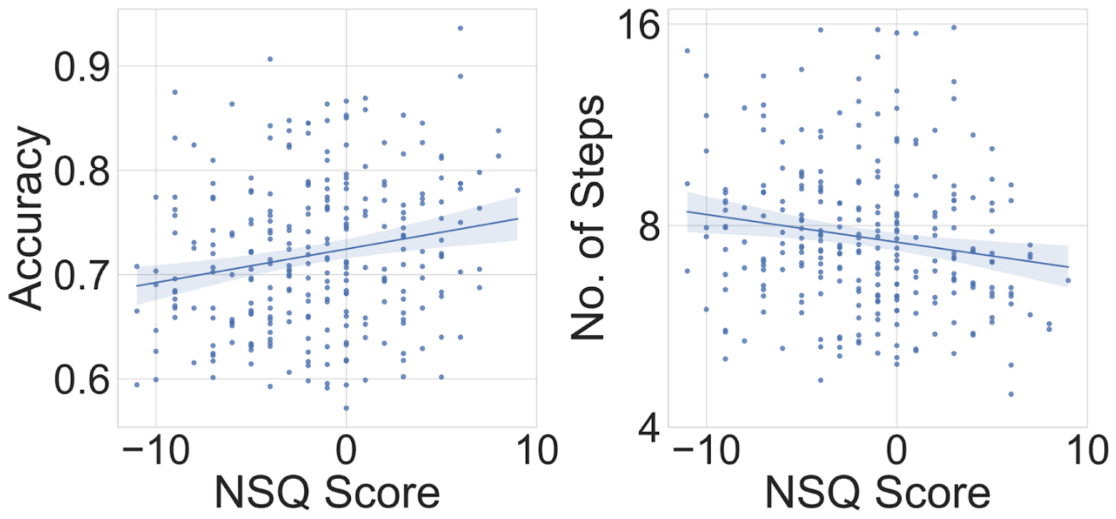

Figure S4: Relationship between scores on the Navigation Strategy Questionnaire (NSQ; x-axis) and performance on the task (y-axis), as measured by accuracy (left) or number of steps to goal (right; presented on a logarithmic scale). Each dot represents and individual participant and the line represents the best-fitting line from a linear regression. Data underlying this figure is available at https://osf.io/w39d5/.

When we pooled data across Experiments 2 and 3 (both of which participants were allowed to freely arbitrate between response strategies throughout the whole experiment, scores on the Navigation Strategy Questionnaire (NSQ), which asked participants about their real-life navigation strategy preferences, were associated with performance (**Fig. S4**). Specifically, higher scores on the NSQ, which indicated a greater preference for map-based (versus landmark-based) strategies, were associated with lower number of steps taken to goal (β = -0.17, *SE* = 0.06, *t*(267) = -2.76, *p* = .006) and accuracy (β = 0.20, *SE* = 0.06, *t*(267) = 3.28, *p* = .001).
