# Supplementary material for: Goal-directed navigation in humans and deep reinforcement learning agents relies on an adaptive mix of vector-based and transition-based strategies
Source: PLoS Biol. 2025 Jul 29;23(7):e3003296. doi: 10.1371/journal.pbio.3003296 (PMC12324678; doi:10.1371/journal.pbio.3003296)
Supplement: S5 Fig — Each dot represents and individual participant and error bars represent the 95% CI. B: accuracy in navigation (y-axis) across the number of landmark conditions (x-axis) before and after a landmark is encountered (hue). Each dot represents an individual participant and error bars represent the 95% CI. C: Proportion of time spent across the whole experiment before encountering a landmark (y-axis) across the number of landmark conditions (x-axis). Each dot represents and individual participant and error bars represent the 95% CI. D: Mean number of landmarks used (i.e., clicked on using a state-based response; y-axis) in the different number of landmark conditions. Each dot represents an individual participant and error bars represent the 95% CI. E: Accuracy in navigation (y-axis) across the number of landmark conditions (x-axis) before and after the edge of the grid is encountered (hue). Each dot represents an individual participant and error bars represent the 95% CI. F: Proportion of time spent on each trial spent on the edge of the grid (y-axis) across the number of landmark conditions (x-axis). Each dot represents an individual participant and error bars represent the 95% CI. Data underlying this figure is available at https://osf.io/w39d5/. (PDF) [file pbio.3003296.s005.pdf]

Supplementary Figure 5: Lack of Effect of Number of Landmarks and the Role of Edges

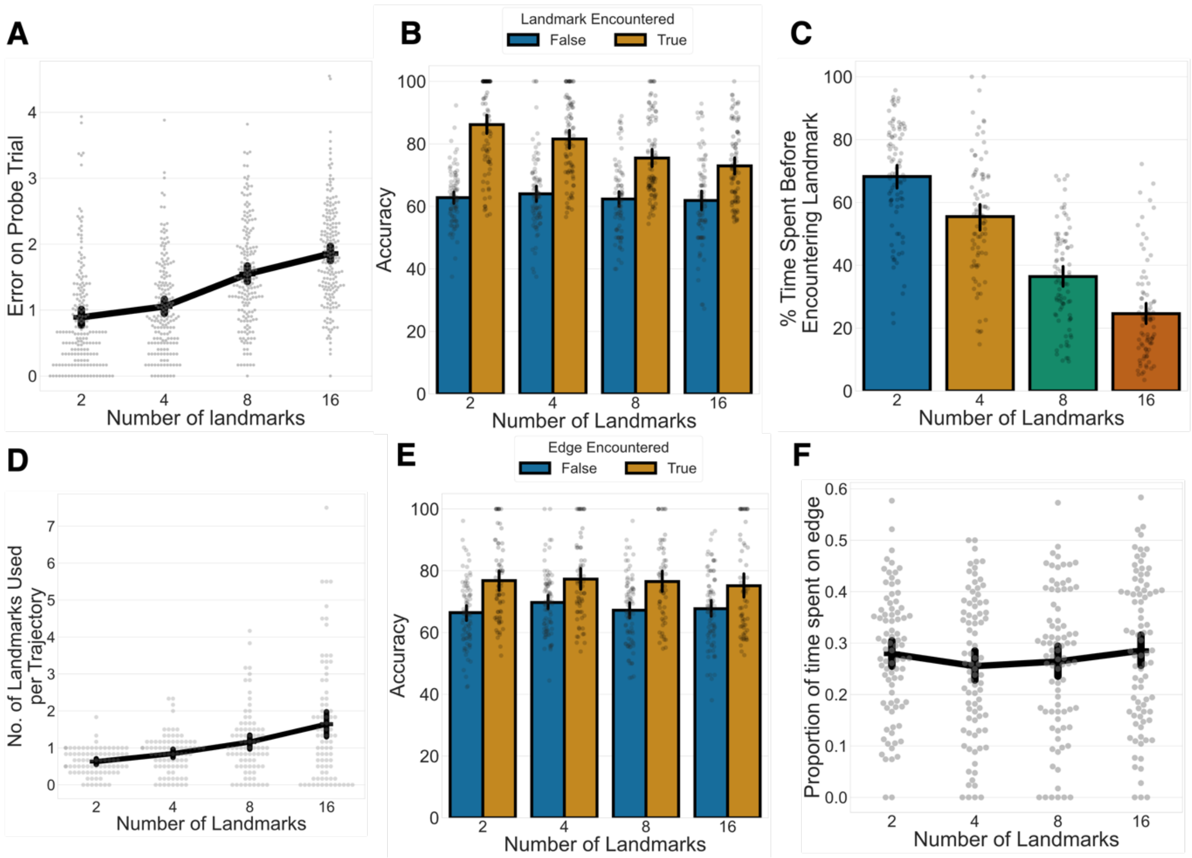

Figure S5: A: Mean error (in Euclidean distance) on memory probe trials for landmarks in each of the landmark number conditions. Each dot represents and individual participant and error bars represent the 95% CI. B: accuracy in navigation (y-axis) across the number of landmark conditions (x-axis) before and after a landmark is encountered (hue). Each dot represents an individual participant and error bars represent the 95% CI. C: Proportion of time spent across the whole experiment before encountering a landmark (y-axis) across the number of landmark conditions (x-axis). Each dot represents and individual participant and error bars represent the 95% CI. D: Mean number of landmarks used (i.e., clicked on using a state-based response; y-axis) in the different number of landmark conditions. Each dot represents an individual participant and error bars represent the 95% CI. E: Accuracy in navigation (y-axis) across the number of landmark conditions (x-axis) before and after the edge of the grid is encountered (hue). Each dot represents an individual participant and error bars represent the 95% CI. F: Proportion of time spent on each trial spent on the edge of the grid (y-axis) across the number of

landmark conditions (x-axis). Each dot represents an individual participant and error bars represent the 95% CI. Data underlying this figure is available at https://osf.io/w39d5/.

When there are fewer landmarks, participants receive more exposures on each landmark and naturally remember the locations of each landmark better. This is evidenced by their performance on memory probe trials in Experiment 3, where participants perform better on probe trials when there are fewer landmarks (**Fig S5A**; ANOVA: $F(3, 552) = 112.95$, $p < .001$). Consequently, the boost in navigation accuracy from encountering a landmark is bigger when there are fewer landmarks (**Fig S5B**; mixed effects model - landmark encountered x number of landmarks interaction: $\beta = -0.046$, $SE = 0.0078$, $z = -5.85$, $p < .001$). However, participants are also naturally less likely to encounter landmarks when there are fewer landmarks overall and spend more time navigating before they encounter their first landmark (**Fig S5C**). The opposing effects of memory and coverage cancel each other, likely accounting for the lack of overall effect.

In any case, it appears that participants do not find the need to use more than one or two landmarks on each trajectory, perhaps limiting the usefulness of having too many additional landmarks. On average, participants in Experiment 2 encountered 2.21 landmarks on each trajectory ($SD = 0.72$), while they actively clicked on (i.e., using a state-based response) only 1.07 landmarks ($SD = 0.65$). Participants are more prone to using more landmarks on each trajectory when there are more available (**Fig S5D**); however, even when there are 16 landmarks, participants only actively use 1.64 landmarks on average.

An alternative to using landmarks for localisation might be using the edges of the grid. For example, participants might realise that they are on the left edge of the grid if they keep moving left and arrive at a boundary. Indeed, people's navigation accuracy improves after encountering the edge of the grid (**Fig. S5E**; logistic mixed effects model - edge encountered: $\beta = 0.34$, SE = 0.068, $z = 5.12$, $p < .0001$). This effect is ever so slightly stronger when there are fewer landmarks (edge encountered x number of landmarks interaction: $\beta = -0.015$, SE = 0.0071, $z = -2.16$, $p = .03$). However, it does not appear to be the case that participants rely more on such a strategy when there are fewer landmarks (**Fig. S5F**), suggesting that reliance on the edges cannot fully account for the lack of difference in the different landmark number conditions.
