# Supplementary material for: Goal-directed navigation in humans and deep reinforcement learning agents relies on an adaptive mix of vector-based and transition-based strategies
Source: PLoS Biol. 2025 Jul 29;23(7):e3003296. doi: 10.1371/journal.pbio.3003296 (PMC12324678; doi:10.1371/journal.pbio.3003296)
Supplement: S6 Fig — Error bars represent the 95% CI. B: Percentage of steps where participants used a landmark (defined as clicking on the image of a landmark on the state-based display; y-axis) as a function of the order in which they passed the landmark on a navigation trial. Error bars represent the 95% CI. C: Same as B but focusing only on the first landmark participants pass by. D: Same as B and C but focusing only on subsequent landmarks that participants pass by. E: Percentage of all landmarks used in the navigation phase (y-axis) that were presented in each serial position in the map-reading phase. Each plot represents a condition with a different number of landmarks. When there were more than 16 landmarks, the same landmarks were presented several times: the lightest hue in each plot indicates the first time the landmarks were presented, with progressively darker hues indicating each subsequent presentation. Error bars represent the 95% CI. Data underlying this figure is available at https://osf.io/w39d5/. (PDF) [file pbio.3003296.s006.pdf]

Supplementary Figure 6: Patterns of Landmark Use

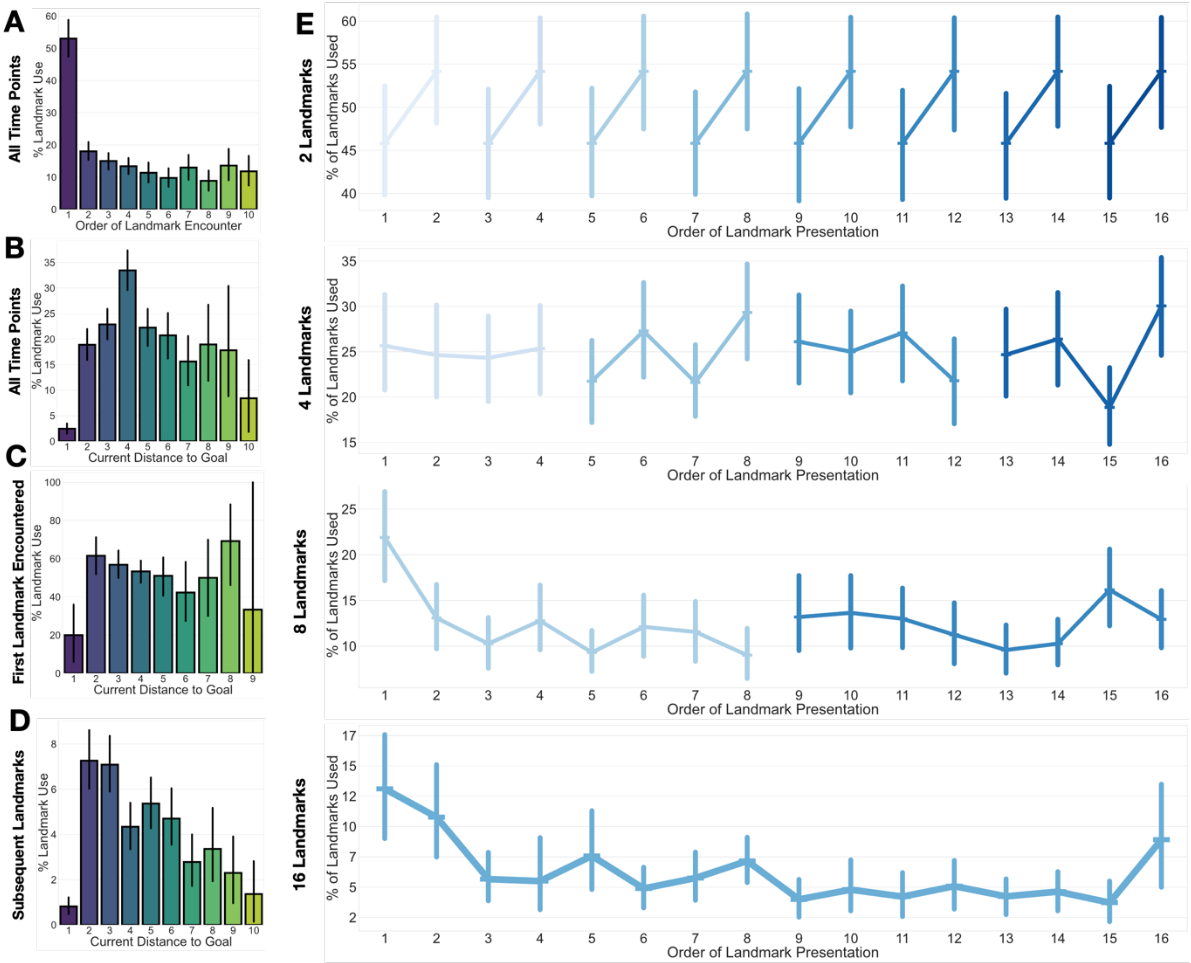

Figure S6: A: Percentage of steps where participants used a landmark (defined as clicking on the image of a landmark on the state-based display; y-axis) as a function of the order in which they passed by the landmark on a navigation trial. Error bars represent the 95% CI. B: Percentage of steps where participants used a landmark (defined as clicking on the image of a landmark on the state-based display; y-axis) as a function of the order in which they passed the landmark on a navigation trial. Error bars represent the 95% CI. C: Same as B but focusing only on the first landmark participants pass by. D: Same as B and C but focusing only on subsequent landmarks that participants pass by. E: Percentage of all landmarks used in the navigation phase (y-axis) that were presented in each serial position in the map-reading phase. Each plot represents a condition with a different number of landmarks. When there were more than 16 landmarks, the same landmarks were presented several times: the lightest hue in each plot indicates the first time the landmarks were presented, with

progressively darker hues indicating each subsequent presentation. Error bars represent the 95% CI. Data underlying this figure is available at https://osf.io/w39d5/.

If participants were trying to minimise uncertainty in their locations as quickly as possible, they might be more likely to use landmarks they encounter early on in their trajectories. Indeed, when participants are adjacent to a landmark, they are significantly more likely to use landmarks when this is the first time they are adjacent to a landmark (**Fig. S6A**). Moreover, it may be interesting to examine whether participants are more likely to use landmarks that are nearer or further to the goal. While ostensibly participants are more likely to use landmarks when they are 4 steps away from the goal compared to when they are closer or further away from the goal (**Fig. S6B**). However, this is likely an artifact of participants starting 4 steps away from the goal, combined with the fact that they are most likely to use the first landmark they encounter. When we plot landmark use separately for the first landmark and the subsequent landmarks participants that encountered (**Fig. S6D/E**), we observe a slight trend towards higher landmark use closer to the goal (with the exception of when participants were adjacent to the goal, when it is more sensible to click on the goal instead).

When we run a logistic mixed effects model predicting landmark use (on timepoints when participants are adjacent to a landmark but not adjacent to a goal), we found that participants were significantly more likely to use landmarks when this was the first landmark they encountered ($\beta = 2.60$, SE = 0.26, $z = 9.95$, $p < .0001$) and slightly less likely to use landmarks when they were further away from the goal ($\beta = -0.06$, SE = 0.030, $z = -1.97$, $p = .049$). There was also an interaction between the two

factors, suggesting that the effect of goal distance was stronger when the landmark was the first landmark they encountered ($\beta = -0.12$, SE = 0.059, $z = -2.03$, $p = .042$).

Given the hypothesised role of memory in landmark use, another interesting question might be whether participants' landmark use is affected by the order in which they were presented in the map-reading phase. We looked across all landmarks used (i.e., where participants had clicked on the image of the landmark) and calculated the proportion of landmarks which had been presented at each serial position in the map-reading phase (**Fig. S6F**). There appeared to be an effect of presentation order in the conditions with more landmarks (8 or 16). Specifically, there appeared to be a primacy effect, where participants were more likely to use landmarks that appeared earlier on in the map-reading phase. We conducted an ANOVA by averaging the proportions for the first 2 ('early'), last 2 ('late'), and middle landmarks and found a significant main effect of order of first landmark presentation on landmark use in the 8 landmark and 16 landmark conditions (8 landmark: $F(2, 148) = 8.70$, $p < .001$; 16 landmark: $F(2, 140) = 9.48$, $p < .001$), while post-hoc t-tests revealed significant differences between the 'early' vs 'middle' and 'early' vs 'late' contrasts, but not the 'middle' vs 'late' contrast in both conditions. The existence of a primacy effect supports the idea that landmark use depends on long-term (possibly hippocampal-dependent) memory of landmark locations. Moreover, the fact that only the first ~2 landmarks are over-represented in participants' landmark use sheds further light on how memory constraints might limit any potential benefits of additional landmarks.
