# Supplementary material for: Goal-directed navigation in humans and deep reinforcement learning agents relies on an adaptive mix of vector-based and transition-based strategies
Source: PLoS Biol. 2025 Jul 29;23(7):e3003296. doi: 10.1371/journal.pbio.3003296 (PMC12324678; doi:10.1371/journal.pbio.3003296)
Supplement: S7 Fig — Each red line represents the training curve for a single model with a single random seed, while the black line represents the mean across all models. (PDF) [file pbio.3003296.s007.pdf]

Supplementary Figure 7: Training Curves

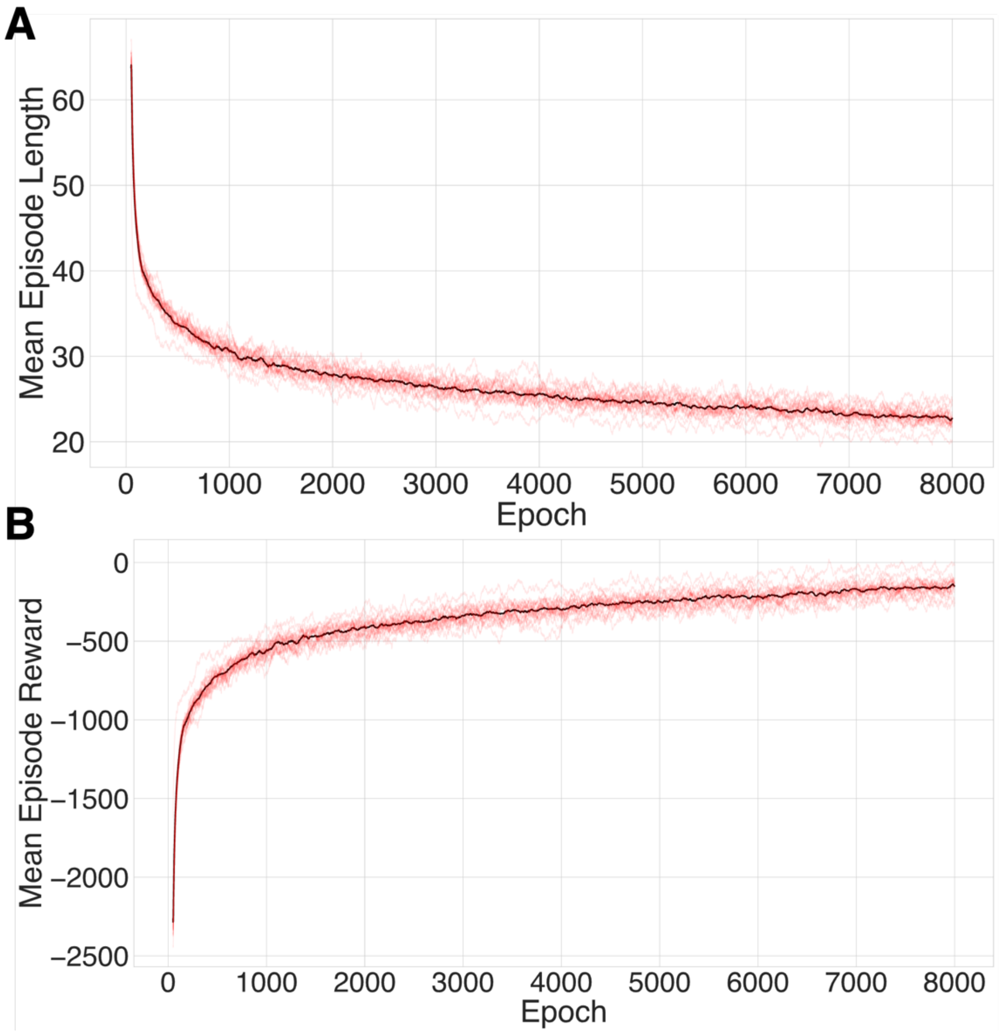

Figure S7: Training curves for deep meta-RL agents showing how (A) mean episode length and (B) reward evolves with training epoch. Each red line represents the training curve for a single model with a single random seed, while the black line represents the mean across all models.
