# Supplementary material for: Goal-directed navigation in humans and deep reinforcement learning agents relies on an adaptive mix of vector-based and transition-based strategies
Source: PLoS Biol. 2025 Jul 29;23(7):e3003296. doi: 10.1371/journal.pbio.3003296 (PMC12324678; doi:10.1371/journal.pbio.3003296)
Supplement: S8 Fig — Each dot represents a model with a different initialization seed, and the error bars represent the 95% CI. B: Same as A but for models with perfect memory of landmarks and goals. C: Models’ use of vector-based responses (y-axis) as a function of destination type (i.e., goal, landmark, or non-landmark; x-axis) and whether the state had been visited before (color of bar). Each dot represents the behavior of an individual model, with error bars representing the 95% CI. D: Same as C but for models with perfect memory of landmarks and goals. F: Mean number of landmarks used (i.e., clicked on using a state-based response; y-axis) in the different number of landmark conditions. Each dot represents an individual model and error bars represent the 95% CI. G: Same as C but for models with perfect memory of landmarks and goals. (PDF) [file pbio.3003296.s008.pdf]

Supplementary Figure 8: Effect of Number of Landmarks and Noisy Memory in Models

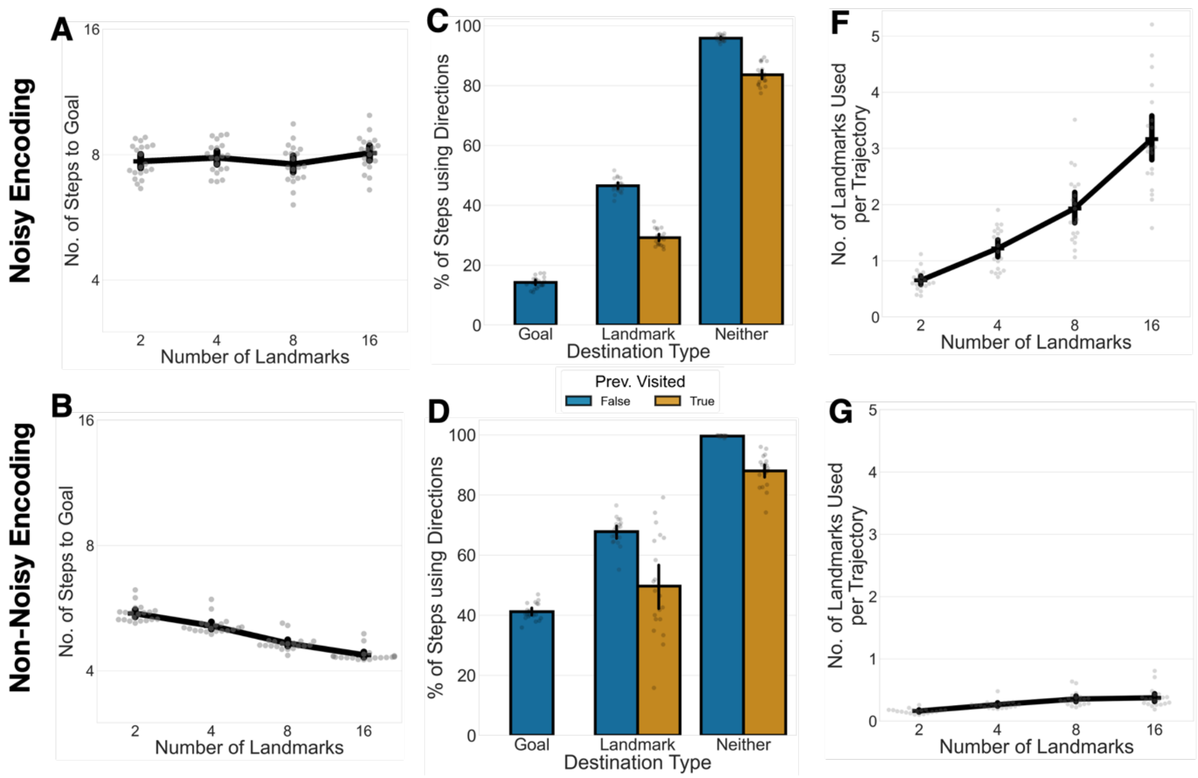

Figure S8: A: Performance as measured by number of steps taken to goal (y-axis; presented on a logarithmic scale) as a function of number of landmarks (x-axis) for models with noisy landmark and goal encodings. Each dot represents a model with a different initialisation seed, and the error bars represent the 95% CI. B: Same as A but for models with perfect memory of landmarks and goals. C: Models' use of vector-based responses (y-axis) as a function of destination type (i.e., goal, landmark, or non-landmark; x-axis) and whether the state had been visited before (colour of bar). Each dot represents the behaviour of an individual model, with error bars representing the 95% CI. D: Same as C but for models with perfect memory of landmarks and goals. F: Mean number of landmarks used (i.e., clicked on using a state-based response; y-axis) in the different number of landmark conditions. Each dot represents an individual model and error bars represent the 95% CI. G: Same as C but for models with perfect memory of landmarks and goals.

As with humans, the performance of models with noisy landmark and goal encodings were not affected by the number of landmarks (**Fig. S8A**). When we run a similar linear mixed effects model as we did in humans (with number of landmarks, coded as a categorical variable with '8' as the reference category) as a predictor of log-transformed steps-to-goal, there were no significant differences between any of the landmark conditions.

In our explanation for why we did not observe an effect of the number of landmarks in humans, we had argued that memory constraints played a key role in the lack of effect. Hence, if a model were trained and tested without noisy landmark and goal encodings (i.e., simulating perfect memory of landmarks and goals), we should observe that performance improves with an increasing number of landmarks. When we re-trained 20 models with perfect goal and landmark encodings, this is indeed what we observe (**Fig. S8B**). In a linear mixed effects model (same as described above), compared to when there were 8 landmarks, models' performance were worse when there were 2 (β = 0.32, SE = 0.068, z = 46.72, $p < .001$) or 4 landmarks (β = 0.18, SE = 0.0054, z = 33.96, $p < .001$), but better when there were 16 landmarks (β = -0.20, SE = 0.0059, z = -33.71, $p < .001$). This supports the idea that memory constraints are a key reason why we do not observe an effect of the number of landmarks in humans and models with noisy landmark and goal encodings.

These simulations also allow us to ask how memory constraints affect models' use of navigation strategies. The main purpose of using a 'state-based' response in our task is for agents to localise themselves in the environment, which in turn allows them

to use ‘direction-based’ responses more effectively. When memory for landmarks is perfect, we might expect that the need for localisation might decrease, and agents would consequently use landmarks less. This turns out to be true (**Fig. S8C** vs **S8D**). Importantly, with noisy landmark encodings, agents might occasionally integrate over multiple landmarks to effectively localize themselves, especially in the high-landmark conditions where the location of each individual landmark is remembered less well. Hence, agents with non-noisy landmark encodings are prone to use multiple landmarks per trajectory, especially when there are more landmarks (**Fig. S8E**, as humans do: **Fig. S5D**). However, agents with perfect landmark encodings end up using markedly fewer landmarks on each trajectory–using on average, less than one landmark on each run (**Fig. S8F**).
