# Supplementary material for: Goal-directed navigation in humans and deep reinforcement learning agents relies on an adaptive mix of vector-based and transition-based strategies
Source: PLoS Biol. 2025 Jul 29;23(7):e3003296. doi: 10.1371/journal.pbio.3003296 (PMC12324678; doi:10.1371/journal.pbio.3003296)
Supplement: S9 Fig — ‘Vector’ units are more likely to have stable spatial responses, while ‘transition’ units are more likely to have remapping spatial responses. (PDF) [file pbio.3003296.s009.pdf]

<u>Supplementary Figure 9: Stability-based Classification of Spatial Cells</u>

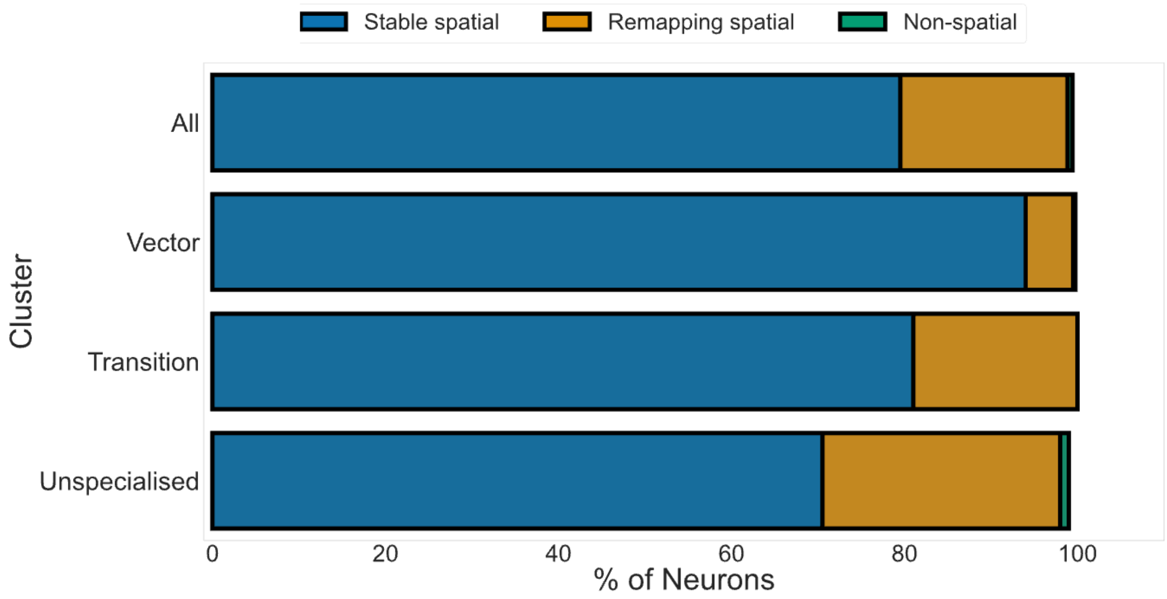

Figure S9: Stacked bar plots showing the proportion of response pattern types ('stable spatial', 'remapping spatial', or 'non-spatial') across all units or in the 'vector', 'transition', or 'unspecialised' clusters. 'Vector' units are more likely to have stable spatial responses, while 'transition' units are more likely to have remapping spatial responses.

We classified 'spatial' cells based on activity over quadrants because it allowed us to easily identify localised spatial firing patterns and how they interacted how these patterns shifted when landmarks were placed in each of the four different quadrants in different environments. However, while our quadrant-based method for classifying spatial cells can detect cells with contiguous firing fields centred mostly within a single quadrant, this method might fail to classify other types of spatial firing patterns. An alternative method for classifying spatial cells involves examining the stability of spatial activation maps across trials and environments. We calculate the linear correlation between spatial activation maps on even timesteps with that of odd timesteps either within the same environment, or in a different environment

(with different landmark positions). As a control, we also compute the correlation between spatial activation maps on even timesteps for each unit with odd trials of all other units, similar to methods for classifying place cells based on stability in electrophysiological studies [1]. We classify cells as 'spatial' if their within-environment, within-cell correlation coefficients were within the 95th percentile of all shuffled within-environment correlation coefficients. Additionally, we classify cells as stable if their cross-environment correlation coefficients were within the 95th percentile of shuffled cross-environment correlation coefficients.

Using this metric, a vast majority of cells (79.50%) were classified as having 'stable spatial' response patterns, with some exhibiting 'remapping spatial' response patterns (19.35%) and a small minority having non-spatial response patterns. The proportion of 'stable spatial' response patterns identified using this metric is substantially larger than our quadrant-based metric—this is expected as this metric imposes less constraints on the nature of these response patterns (e.g., peak responses need not be confined within specific quadrants). However, when we looked at the relationship between spatial response patterns and the functional classification across units, we found a similar significant association between response pattern and functional classification ($\chi 2$ = 40.38, d.f. = 4, $p < .0001$, n = 600). 'Vector' units were significantly less likely to have a 'remapping' spatial response (Adjusted Pearson residual = -4.02, $p < .0001$). This is in line with the idea that 'vector' units exploit generalisable spatial information across environments, while 'transition' units might be more likely to remap across different environmental contexts.

References:

1. Grijseels DM, Shaw K, Barry C, Hall CN. Choice of method of place cell classification determines the population of cells identified. PLOS Comput Biol. 2021;17: e1008835. doi:10.1371/journal.pcbi.1008835
