# Supplementary material for: Goal-directed navigation in humans and deep reinforcement learning agents relies on an adaptive mix of vector-based and transition-based strategies
Source: PLoS Biol. 2025 Jul 29;23(7):e3003296. doi: 10.1371/journal.pbio.3003296 (PMC12324678; doi:10.1371/journal.pbio.3003296)
Supplement: S10 Fig — ‘Gridness’ scores are shown on top of each unit’s spatial autocorrelogram. (PDF) [file pbio.3003296.s010.pdf]

Supplementary Figure 10: 'Gridness' Scores

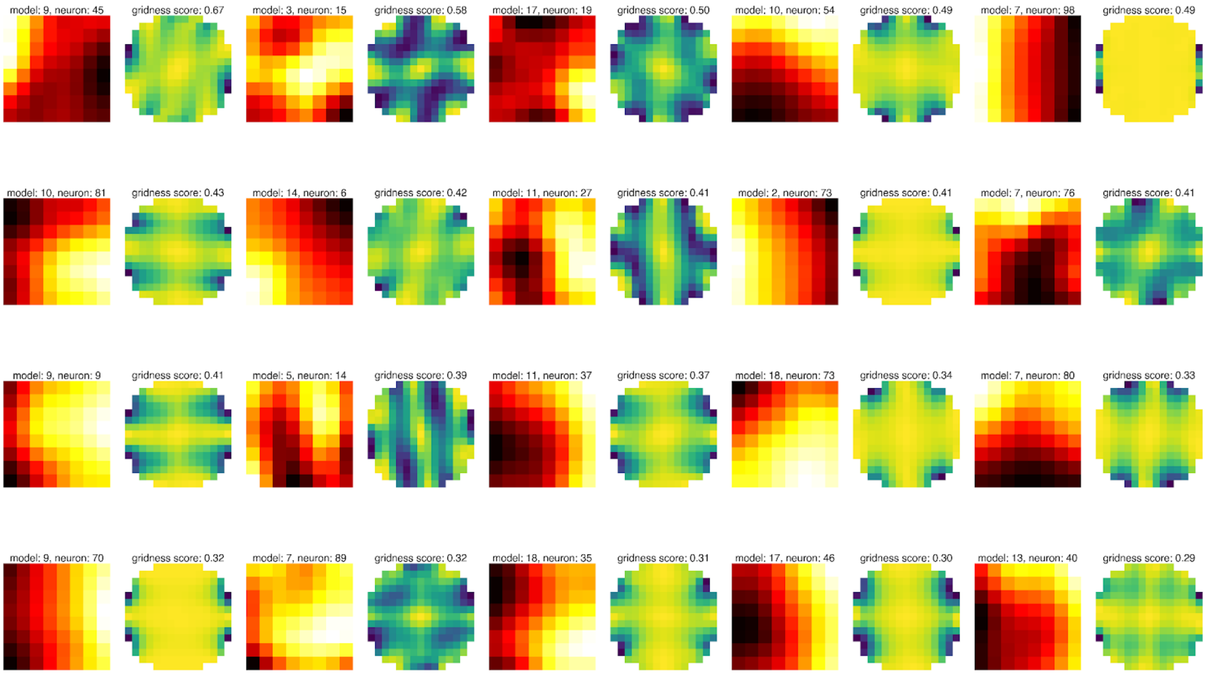

Figure S10: Sample of activation heatmaps (left) and spatial autocorrelograms (right) for units with the highest 'gridness' scores. 'Gridness' scores are shown on top of each unit's spatial autocorrelogram.

Grid cells have been proposed to underlie vector-based navigation [1,2]. Since the networks appear to learn to successfully use vector-based strategies, we asked whether we could identify emergent grid-like representations in the networks.

When we apply traditional measures for 'gridness' scoring (using code adapted from Banino et al. [2]), we do identify a couple units that ostensibly fit the criteria for 'grid cells'. In other words, these cells have spatial autocorrelograms that exhibit 60 degree rotational symmetry within the 95th percentile null distribution (generated from field-shuffled activation maps). On the surface, these so-called 'grid' cells are more commonly found amongst the 'vector' units than the 'transition' and 'unspecialised' units ($\chi 2$ = 7.20, d.f. = 4, p = .03, n = 600), consistent with their suggested role in

vector-based computations [2]. However, an inspection of the actual activation patterns of these supposed ‘grid cells’ suggests that these patterns are far from grid-like (**Fig. S10**). This is an established problem when applying traditional ‘gridness’ scoring methods, especially on responses from artificial neural networks: while ‘gridness’ scores focus on rotational symmetry and grid cells indeed have rotational symmetry, activation maps may also exhibit symmetry without necessarily being grid-like [3]. In any case, the gridness scores we get are markedly lower than those identified in animals and in other simulations involving neural networks.

Importantly, our models have several important differences from previous studies that have purportedly identified grid cells in task-trained neural networks. In the first place, the networks in previous studies and in our study are trained on vastly different tasks. Most previous studies have trained recurrent networks on path integration tasks which attempt to mimic rodents freely roaming around an arena, often receiving (often two-dimensional) velocity vectors as inputs. In contrast, our networks were trained on a task where they were only allowed to move in the four cardinal directions around a square grid. There was no concept of velocity in the task (as networks always moved one square per timestep), and instead of velocity vectors, networks received as input information about landmarks.

Crucially, the networks in most previous studies were trained either with supervised objectives (e.g., to predict their current location) [2,4,5], or in more recent studies, with self-supervised objectives such as maintaining path invariance and separation between locations [6] or predictive coding [7]. In contrast, our networks were trained to maximise reward on our task (i.e., with a reinforcement learning objective). To our

knowledge, no previous studies have identified grid cells in networks trained using only a reinforcement learning objective.

Many previous modelling works have involved navigation in continuous environments, whereas our models were trained to navigate in a grid-world. A notable model that has discovered grid codes in agents trained in grid environments is the Tolman-Eichenbaum Machine (TEM) [8]. However, TEM also has many differences from our networks. For example, TEM is trained with a predictive objective (vs. a reward-maximisation objective in our networks), has a vastly different network architecture (i.e., explicitly factorising structural and sensory codes and representing their conjunction through Hebbian weights), and has other built-in biological constraints (e.g., non-negative firing).

Moreover, in previous studies, the emergence of grid cells often depend on particular constraints and inductive biases built into the network, such as non-negativity [6] or particular target encodings on supervised tasks (e.g., Difference-of-Gaussian read-outs) [2,5]. These constraints appear critical to the emergence of grid cells, and networks trained on path-integration tasks without these constraints almost never develop grid cells [3], suggesting that a path-integration objective alone is not sufficient for the emergence of grid cells. We trained our networks without any of these constraints, and it might hence be unsurprising that we do not observe grid cells.

Overall, the absence of grid-like representations in our networks is particularly notable given the importance of vector-based navigation in our task. Evidently,

learning to solve navigation tasks requiring vector-based strategies alone is not sufficient for grid-like codes to develop. This hence raises an interesting question: why do brains use grid codes for vector-based navigation when vector-based navigation can be achieved without grid-like representations? Future work should more carefully examine the conditions that may be sufficient for grid-like codes to emerge, which may be specific training objectives (e.g., supervised or predictive losses), biological constraints (e.g., non-negativity), or environments (e.g., continuous metric spaces vs grid-worlds).

References:

1. Bush D, Barry C, Manson D, Burgess N. Using Grid Cells for Navigation. Neuron. 2015;87: 507–520. doi:10.1016/j.neuron.2015.07.006

2. Banino A, Barry C, Uria B, Blundell C, Lillicrap T, Mirowski P, et al. Vector-based navigation using grid-like representations in artificial agents. Nature. 2018;557: 429–433. doi:10.1038/s41586-018-0102-6

3. Schaeffer R, Khona M, Fiete IR. No Free Lunch from Deep Learning in Neuroscience: A Case Study through Models of the Entorhinal-Hippocampal Circuit. 2022. Available: https://openreview.net/forum?id=syU-XvinTI1

4. Cueva CJ, Wei X-X. Emergence of grid-like representations by training recurrent neural networks to perform spatial localization. arXiv; 2018. doi:10.48550/arXiv.1803.07770

5. Sorscher B, Mel GC, Ocko SA, Giocomo LM, Ganguli S. A unified theory for the computational and mechanistic origins of grid cells. Neuron. 2023;111: 121-137.e13. doi:10.1016/j.neuron.2022.10.003

6. Schaeffer R, Khona M, Ma T, Eyzaguirre C, Koyejo S, Fiete IR. Self-Supervised Learning of Representations for Space Generates Multi-Modular Grid Cells. arXiv; 2023. doi:10.48550/arXiv.2311.02316

7. Tang M, Barron H, Bogacz R. Learning grid cells by predictive coding. arXiv; 2024. doi:10.48550/arXiv.2410.01022

8. Whittington JCR, Muller TH, Mark S, Chen G, Barry C, Burgess N, et al. The Tolman-Eichenbaum Machine: Unifying Space and Relational Memory through Generalization in the Hippocampal Formation. Cell. 2020;183: 1249-1263.e23. doi:10.1016/j.cell.2020.10.024
