# Supplementary material for: Goal-directed navigation in humans and deep reinforcement learning agents relies on an adaptive mix of vector-based and transition-based strategies
Source: PLoS Biol. 2025 Jul 29;23(7):e3003296. doi: 10.1371/journal.pbio.3003296 (PMC12324678; doi:10.1371/journal.pbio.3003296)
Supplement: S11 Fig — Each dot represents the centroid of the PCs for each location in the grid. In the left plots (A, B, and C), red and blue dots represent the PCs before and after a landmark is encountered, respectively. In the right plots (D, E, and F), green and purple dots represent the PCs at a landmark or a non-landmark. PCA results are only shown for one representative model. (PDF) [file pbio.3003296.s011.pdf]

Supplementary Figure 11: Representational Geometry of Meta-Learning Agent

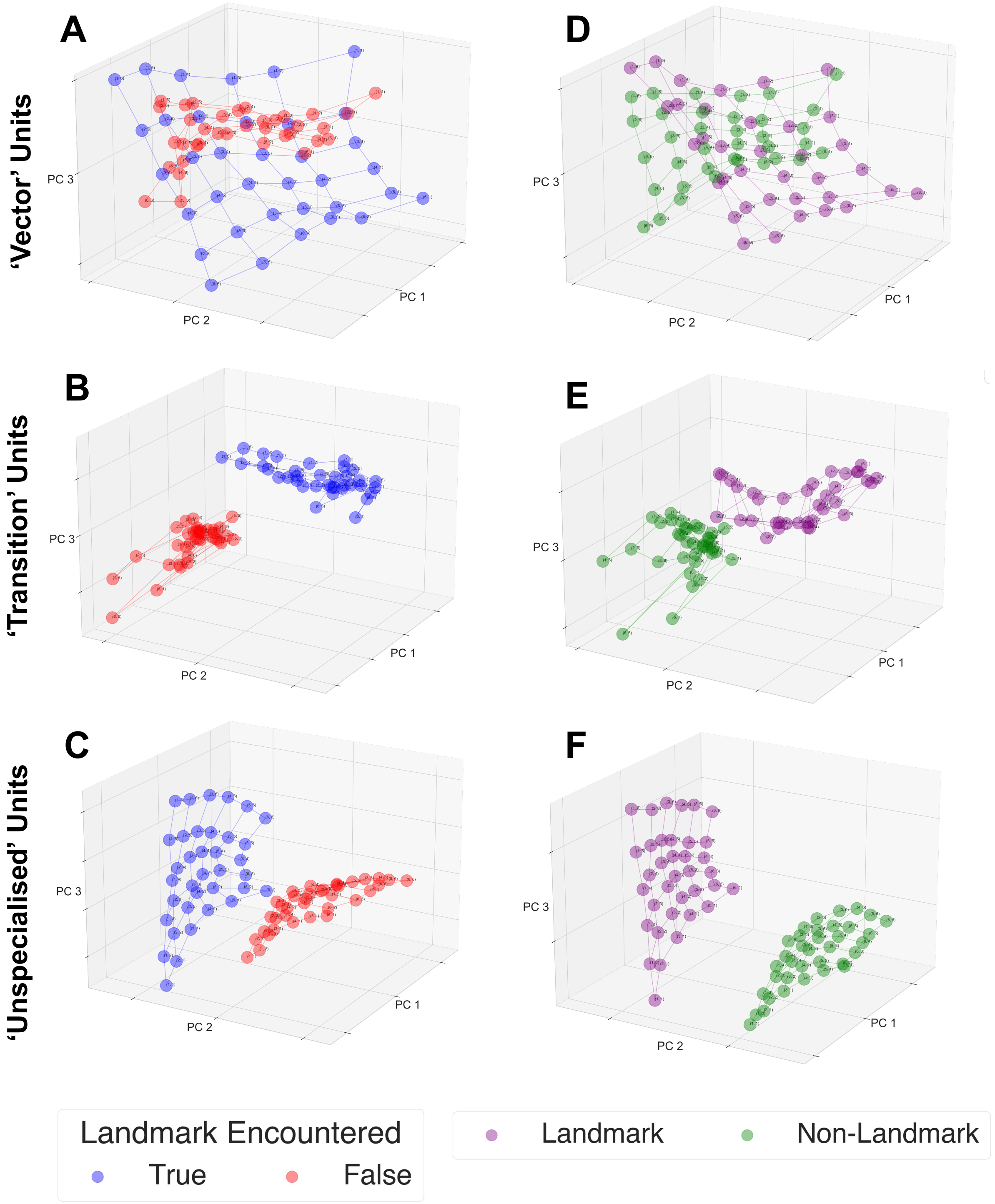

Figure S11: First three principal components for the PCA on the cell state activations of 'vector' units (A and D), 'transition' units (B and E), and 'unspecialised' units (C and F). Each dot represents the centroid of the PCs for each location in the grid. In the left plots (A, B, and C), red and blue dots

represent the PCs before and after a landmark is encountered respectively. In the right plots (D, E, and F), green and purple dots represent the PCs at a landmark or a non-landmark. PCA results are only shown for one representative model.
