# Supplementary material for: Goal-directed navigation in humans and deep reinforcement learning agents relies on an adaptive mix of vector-based and transition-based strategies
Source: PLoS Biol. 2025 Jul 29;23(7):e3003296. doi: 10.1371/journal.pbio.3003296 (PMC12324678; doi:10.1371/journal.pbio.3003296)
Supplement: S12 Fig — Each dot represents the centroid of the PCs for each location in the grid. Red and blue dots represent the PCs before and after a landmark is encountered, respectively. PCA results are only shown for one representative model. B: First three principal components for the PCA on the cell state activations of ‘transition’ units. Each dot represents the centroid of the PCs for each location in the grid. Green and purple dots represent the PCs for non-landmarks and landmarks, respectively. The representations of ‘transition’ units seem to separate landmarks and non-landmarks without apparent spatial structure. Note that the edges are not represented for landmarks because the task did not allow landmarks to be placed on the edges. (PDF) [file pbio.3003296.s012.pdf]

Supplementary Figure 12: Representational Geometry of Meta-Learning Agent, with Edges

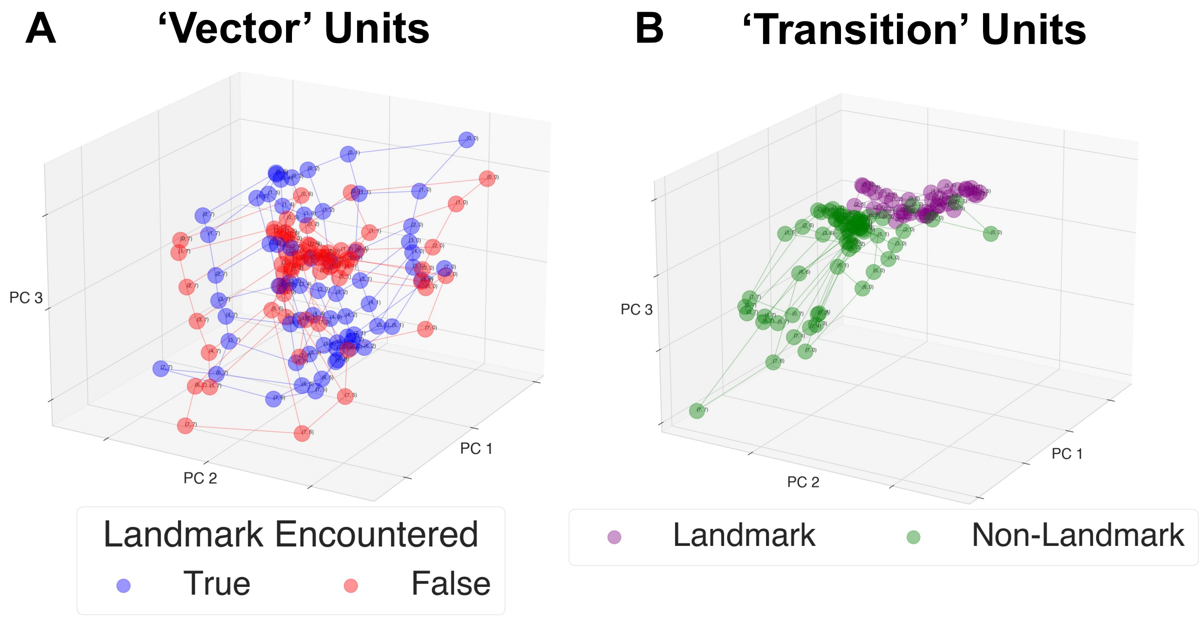

Figure S12: A. First three principal components for the PCA on the cell state activations of 'vector' units. Each dot represents the centroid of the PCs for each location in the grid. Red and blue dots represent the PCs before and after a landmark is encountered respectively. PCA results are only shown for one representative model. B. First three principal components for the PCA on the cell state activations of 'transition' units. Each dot represents the centroid of the PCs for each location in the grid. Green and purple dots represent the PCs for non-landmarks and landmarks respectively. The representations of 'transition' units seem to separate landmarks and non-landmarks without apparent spatial structure. Note that the edges are not represented for landmarks because the task did not allow landmarks to be placed on the edges.

The representational geometries of both 'vector' and 'transition' units appear to be distorted at edges. This is likely because the edges of the grid are special in several ways. For example, there were less possible actions at the edges, the optimal actions

at edges often were to simply move away from the edge, and the task did not allow landmarks and goals to appear on the edges. Importantly, edges also allowed the agent to localise themselves without the use of landmarks. For instance, if the agent moved right until it encountered an edge, it could infer that it was now on the right edge of the grid. In other words, being on the edge reduced the need for landmarks for self-localisation. In the ‘vector’ units, this might be why the centroids for the edges do not appear to be different before and after a landmark was encountered, even though the centroids for every other location appear to change substantially after a landmark was encountered (**Fig. S12A**).
