# Supplementary material for: Goal-directed navigation in humans and deep reinforcement learning agents relies on an adaptive mix of vector-based and transition-based strategies
Source: PLoS Biol. 2025 Jul 29;23(7):e3003296. doi: 10.1371/journal.pbio.3003296 (PMC12324678; doi:10.1371/journal.pbio.3003296)
Supplement: S13 Fig — The two configurations of four landmarks that appear to be represented by the second exploration principal component minimize the mean distance to the nearest landmark. C: Correlation plots representing the relationship between the three exploration-related principal components and the three hypothesis-driven metrics (mean distance to nearest landmark, mean distance to center, and mean error on probe trials). In the plots for the mean distance to the nearest landmark (first column), the hues represent a median split in the mean number of unique landmarks chosen by participants. Blue represents participants who chose a lower number of unique landmarks, while orange represents participants who chose a higher number of unique landmarks. (PDF) [file pbio.3003296.s013.pdf]

Supplementary Figure 13: Relationship between Exploration PCs and Hypotheses-Driven Metrics

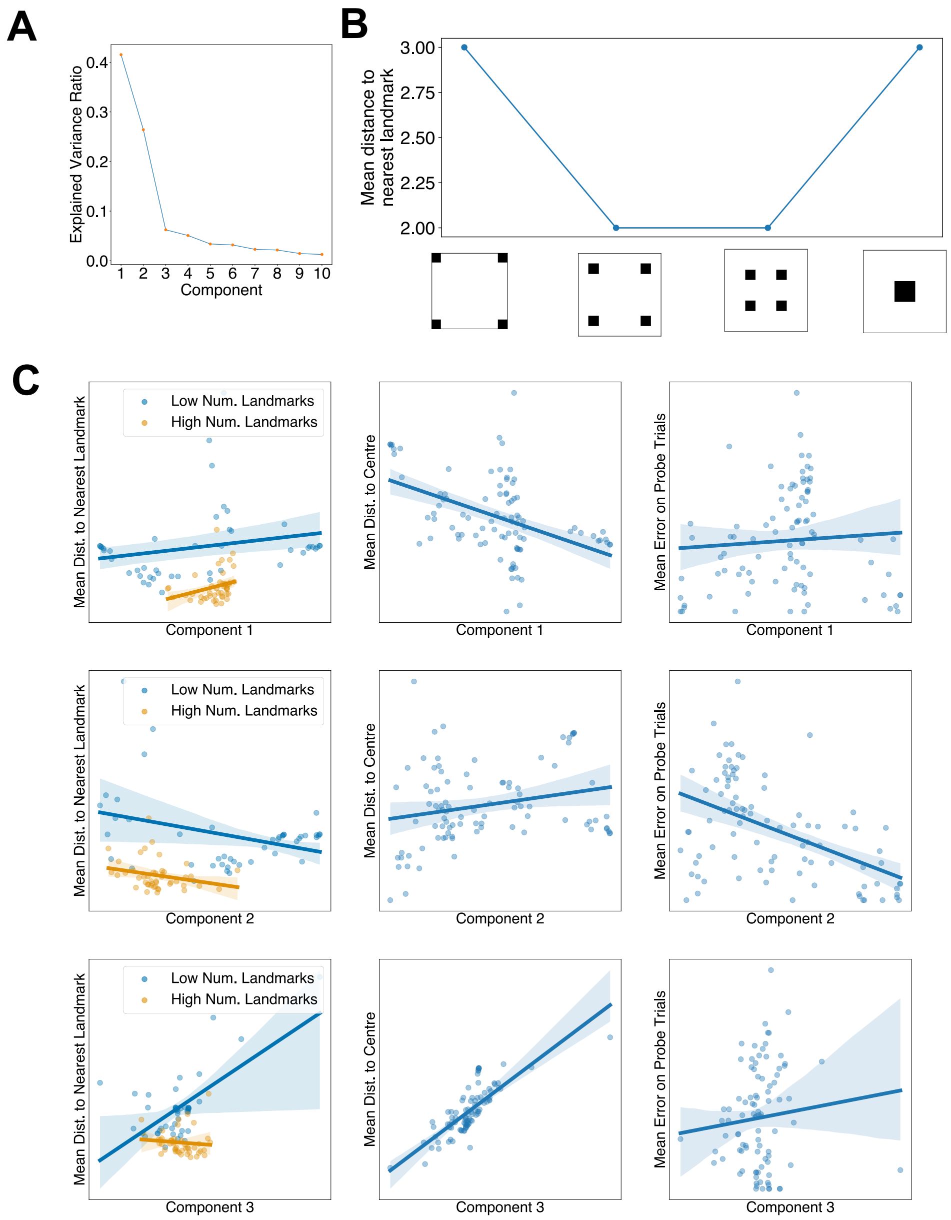


*Figure S13:* A. Plot showing the variance explained by each exploration component. B. Graph illustrating four different configurations of four landmarks and their relationship with the mean distance between all locations and their nearest landmark. The two configurations of four landmarks that appear to be represented by the second exploration principal component minimise the mean distance to the nearest landmark. C. Correlation plots representing the relationship between the three exploration-

related principal components and the three hypothesis-driven metrics (mean distance to nearest landmark, mean distance to centre, and mean error on probe trials). In the plots for the mean distance to the nearest landmark (first column), the hues represent a median split in the mean number of unique landmarks chosen by participants. Blue represents participants who chose a lower number of unique landmarks, while orange represents participants who chose a higher number of unique landmarks.

After controlling for the number of unique landmarks, the average distance from all squares to the nearest landmark was significantly predicted by the first exploration PC ($\beta$ = -0.0017, *SE* = 0.001, $t(87) = 3.30$, $p = .0001$), the second exploration PC ($\beta$ = -0.0068, *SE* = 0.001, $t(87) = -7.78$, $p < .0001$), and the third exploration PC ($\beta$ = -0.010, *SE* = 0.001, $t(87) = 7.65$, $p < .0001$). We controlled for the number of landmarks in the linear regression model as having more landmarks reduces the mean distance to the nearest landmark regardless of their configuration. Without controlling for the number of unique landmarks, exploration PC1 and 2 are no longer significant predictors of mean distance to nearest landmark as they relate to particular strategies for arranging a limited number of landmarks.

All three exploration components also predicted the mean distance between all landmarks and the centre of the grid. Landmark centrality was significantly predicted by exploration PC1 ($\beta$ = -0.0055, *SE* = 0.0004, $t(87) = -12.37$, $p < .0001$), exploration PC 2 ($\beta$ = -0.0036, *SE* = 0.001, $t(87) = 6.36$, $p < .0001$), and exploration PC 3 ($\beta$ = 0.025, *SE* = 0.001, $t(87) = 21.31$, $p < .0001$).

After controlling for the mean error for the probe trials on the first day, the mean error for probe trials on the second day was significantly predicted by exploration PC2 ($\beta$

= -0.0082, *SE* = 0.002, *t*(87) = -4.35, *p* < .0001). This appeared to be mediated by the number of unique landmarks, as the correlation was no longer significant after including the number of unique landmarks as an additional predictor variable, and the number of unique landmarks was instead a significant predictor of mean error on memory probe trials ($\beta$ = 0.15, *SE* = 0.023, *t*(87) = 6.72, *p* < .0001). Indeed, the number of unique landmarks were significantly predicted by exploration PC2 ($\beta$ = -0.058, *SE* = 0.007, *t*(87) = -8.27, *p* < .0001), such that participants who scored higher on exploration PC2 tended to have selected fewer unique landmarks.
