# Supplementary material for: Goal-directed navigation in humans and deep reinforcement learning agents relies on an adaptive mix of vector-based and transition-based strategies
Source: PLoS Biol. 2025 Jul 29;23(7):e3003296. doi: 10.1371/journal.pbio.3003296 (PMC12324678; doi:10.1371/journal.pbio.3003296)
Supplement: Attachment — Submitted filename: Response to Reviewers.pdf [file pbio.3003296.s016.pdf]

Response to Reviewers:

Thank you for the opportunity to revise our manuscript. We sincerely appreciate the editor and reviewers for time and effort invested into providing thoughtful and constructive feedback on our work.

In our response below, reviewers' comments are reproduced in black and our response is presented in blue. We have performed several new analyses and made several edits based on the reviewers' comments and suggestions, which we believe have substantially improved the quality of our manuscript. These new analyses and changes are highlighted in **bold**. The line numbers cited in this response correspond to the 'tracked changes' version of the manuscript.

Reviewer #1: The authors present the results of three online behavioural studies that examine the strategies used to support 'few-shot' goal-directed navigation in novel environments. Specifically, they contrast the use of topological / transition-based strategies and geometric / vector-based strategies using a task in which participants have to navigate around an 8 x 8 grid, wherein each location is either marked by a unique visual 'landmark' or contains an obstacle. Participants begin by seeing an overhead view of the map without any visible landmarks, and are prompted to click on between 2 and 16 locations to reveal the visual landmark therein, before being prompted to click on the goal location. They are then placed at a random start location and must choose between two navigation strategies for each move - either selecting a visual landmark to move to (from those immediately adjacent to their current location), or a movement direction to move in.

In Experiment 1, the authors demonstrate that performance is best when participants can make use of either strategy, rather than being limited to one or the other on each turn; that a vector based strategy is less useful in cluttered environments; and that participants who tended to rely on a single strategy (when both were available) performed poorly. In Experiment 2, the authors demonstrate that the number of landmarks viewed prior to navigation has no effect on performance; and that transition and vector based strategies are preferentially associated with landmark / conjunctive and stable spatial responses in a deep learning agent, respectively. In Experiment 3, participants were given the option to choose which landmarks to view prior to navigation, which led to better performance. This was driven by selecting landmarks that were well distributed across the environment and close to the centre.

The manuscript is clearly written, the experimental task is novel, the analyses are thorough, and these results may be of interest to the field. My only concern is what we can conclude from these findings - i.e. what do they tell us about real world navigation? My concern is that this stylised task bears little resemblance to real world navigation, and so it is not clear what can be concluded or extrapolated from these findings. These concerns are described in more detail below. Given the content, I also wonder if this manuscript is suitable for publication in PLoS Biology? Would these results not be more suitable for

PLoS Computational Biology, given the focus on comprehensive analysis of behaviour and deep meta-RL models?

We thank the reviewer for their positive comments on our manuscript and have attempted to address some of their concerns on ecological validity in our response below. Despite our use of computational models, we believe that our results have broad implications for our understanding of the computational and neural underpinnings of flexible navigation in humans and other animals, and would hence be of interest to the broad readership of PlOS Biology.

Major Concerns

It is not immediately clear what we can conclude from these findings, because I am not sure how ethologically valid this task is.

Our main question concerned the use of state-based and vector-based strategies for navigation, and how human policies related to those of a deep RL model. To test this rigorously, it was necessary to create an experimental situation in which we could manipulate participants' opportunity to navigate with either (1) transitions or (2) directions. We could only do this in a stylised task like the one proposed here. In a more naturalistic setting, we could perhaps attempt to reverse-infer the strategy being at each point in time (and we discussed this) but it seems like this would be prohibitively hard in most environments, and would significantly degrade our confidence in our conclusions. Every experiment designer faces a trade off between experimental control and ecological validity; we opted, in this case, to prioritise the former, and we stand by this decision. However, **in response to these concerns** (and Reviewer 2's comments), **we have extended our discussion on the potential differences between our stylised task and navigation in naturalistic settings** (lines 776 - 782).

Is seems odd to be unaware of one's starting location when navigating, and similarly, to be unaware of the relative location (or direction) of landmarks as you pass them.

It is true that total unawareness of one's location (and the relative location of landmarks) is not the norm for everyday navigation. However, our focus was not on understanding navigation in familiar, overlearned settings, but on navigation in novel environments. In such cases, being unable to orient is much more common. For example, when visiting a new city or exploring a new hiking trail, we might wander around for too long and lose track of our current location. We might also lose track of facing direction, hence becoming unaware of how the landmarks that surround us map onto the cardinal directions. Nevertheless, even then, we might still be able to find our way to our intended destinations reasonably well, perhaps by using a strategy similar to the ones participants use in our stylised task (i.e., heading in the general goal direction, while identifying landmarks we are familiar with that help us localise ourselves in the environment).

I find it worrying that the number of landmarks viewed before navigation began had no impact on performance (Fig 2D).

We share this concern. A priori, we predicted that two factors would mediate how the number of landmarks impacted performance: (1) fewer landmarks offer less opportunity to anchor position in the map; but (2) more landmarks are harder to remember, potentially causing confusion. Imagine you are perusing a map before going hiking - at the extremes, there are potentially detrimental policies (attempting to memorise the entire map, or not paying attention to landmarks at all). We predicted that an intermediate number of landmarks would facilitate navigation. In the event, this was not the case. We interpret the lack of effect as occurring because these two factors tend to compensate for one another, trading off to produce no effect, at least for the quantity of data that we were able to collect.

To address concerns about the lack of effect, we now include some additional analyses looking into the reasons behind the lack of effect. In our task set-up, participants received the same total number of exposures no matter how many landmarks there were. Hence, there were competing costs and benefits for having more or less landmarks in the 'map-reading' phase. When there are fewer landmarks, participants receive more exposures on each landmark and naturally remember the locations of each landmark better. This is evidenced by their performance on memory probe trials in Experiment 3, where participants perform better on probe trials when there are fewer landmarks (Fig. R1A; ANOVA: $F(3, 552) = 112.95$, $p < .001$). Consequently, the boost in navigation accuracy from encountering a landmark is bigger when there are fewer landmarks (Fig R1B; mixed effects model - landmark encountered x number of landmarks interaction: $\beta = -0.046$, SE = 0.0078, $z = -5.85$, $p < .001$). However, participants are also naturally less likely to encounter landmarks when there are fewer landmarks overall, and spend more time navigating before they encounter their first landmark (Fig. R1C). The opposing effects of memory and coverage cancel each other, likely accounting for the lack of overall effect.

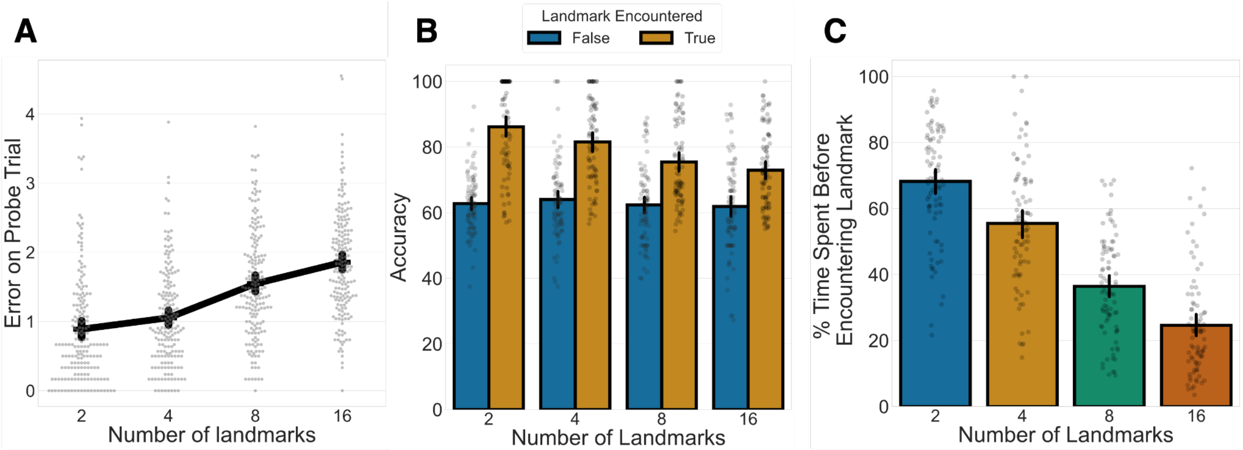

Fig R1: *A: Mean error (in Euclidean distance) on memory probe trials for landmarks in each of the landmark number conditions. Each dot represents and individual participant and error bars represent the 95% CI. B: accuracy in navigation (y-axis) across the number of landmark conditions (x-axis) before and after a landmark is encountered (hue). Each dot represents an individual participant and error bars represent the 95% CI. C: Proportion of time spent across the whole experiment before encountering a landmark (y-axis) across the number of landmark conditions (x-axis). Each dot represents and individual participant and error bars represent the 95% CI.*

In our response to Reviewer 3 below, we also show that the performance of our original models (with noisy memory of landmark and goal locations) also does not change with different numbers of landmarks, but models with perfect memory perform better when there are more landmarks (Fig. R8). This further supports the idea that memory constraints are a key reason why we do not observe an effect.

Again, it is not inconceivable that similar principles might operate in real life: if we have very limited time to study a map before we set off, we can choose to study the locations of a few landmarks very well or to study many landmarks cursorily, both of which might be equally good for performance.

In response to these concerns, **in our revised paper, we have added these analyses plots into the Appendix (Fig. S5) and an explanation for the lack of effect in the main text**: *"The lack of effect of the number of landmarks was likely due to a trade-off between having more landmarks throughout the environment and being able to remember each individual landmark well. Indeed, participants were naturally more likely to encounter a landmark early when there were more landmarks, but participants' navigational accuracy improved more after encountering landmarks when there were fewer landmarks to remember (Fig. S5)."* (lines 328-333)

Overall, my concern is that the authors have very accurately characterised performance on this task, but told us little about navigational strategies in the real world. The authors allude to this possibility in the Discussion. Can they point to anything in the data that validates these findings? For example, did performance correlate with scores on either of the questionnaires that participants completed?

Thanks for suggesting this analysis. **To address the question, we now describe new analyses looking into the relationship between questionnaire measures and performance of our task.** When we pooled data across Experiments 2 and 3 (both of which participants were allowed to freely arbitrate between response strategies throughout the whole experiment, scores on the Navigation Strategy Questionnaire (NSQ), which asked participants about their real-life navigation strategy preferences, were associated with performance (Fig. R2). Specifically, higher scores on the NSQ, which indicated a greater preference for map-based (versus landmark-based) strategies, were associated with lower number of steps taken to goal ($\beta = -0.17$, SE = 0.06, $t(267) = -2.76$, $p = .006$) and accuracy ($\beta = 0.20$, SE = 0.06, $t(267) = 3.28$, $p = .001$).

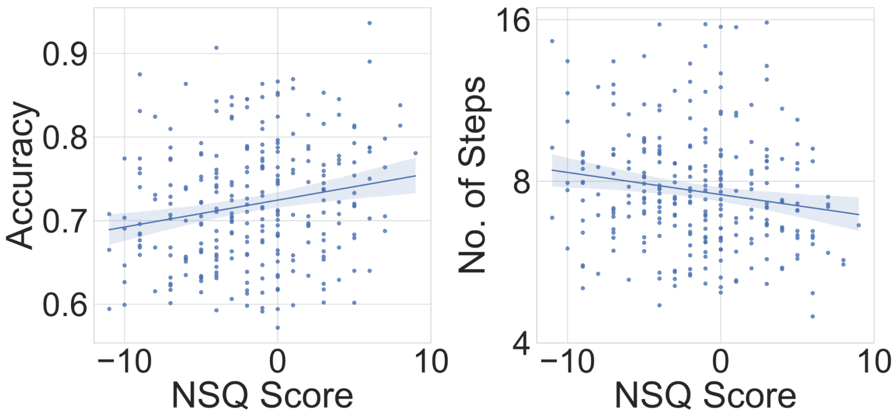

Fig R2: *Relationship between scores on the Navigation Strategy Questionnaire (NSQ; x-axis) and performance on the task (y-axis), as measured by accuracy (left) or number of steps to goal (right; presented on a logarithmic scale). Each dot represents and individual participant and the line represents the best-fitting line from a linear regression.*

While the stylized nature of our task was essential for dissociating vector-based and state transition-based strategies during navigation, it is perhaps reassuring that there is at least some correlation between task performance and participants' self-reported navigation strategies in real-life contexts. **In our revised paper, we have added these plots in the Appendix (Fig. S4)** for readers who might be curious about relationships between questionnaire measures and behaviour on our task.

The task could be explained more clearly in the main text, as this is crucial to understand the results that follow. Specifically, it would be useful to mention that: (i) during the map reading phase, participants have a single forced choice of which (blue) square to click on, and these landmark images remain on screen for <3s; (ii) start locations were always four steps away from the goal location; and that (iii) one of the adjacent locations could be marked with a boulder (i.e. obstacle), in which case clicking on this image (or the corresponding direction) would have no effect. It would also be useful to clarify whether there were any repeats of landmark stimuli across trials ("Every trial involved a grid with completely different sets of images…")?

Thank you for the suggestions - we have **implemented these changes in the main text (lines 128 - 156)** to improve the clarity of the task description.

Minor Comments

Several references are repeated (i.e. 8 and 9, 10 and 32, 24 and 29, 27 and 30 etc)

Thank you for pointing this out - this has been **fixed**.

It is confusing to switch between 'obstacles' and 'clutter locations' - the use of a single term would be clearer

We have **changed 'clutter locations' to *'obstacle locations'*** to maintain consistency.

Figure 2D: It seems odd to me that participants would ever use a directional strategy to reach the goal when they are adjacent to it (and it can therefore be seen as one of the four landmark options) - can the authors offer any explanation of why this might be?

When participants were extremely confident in their location in the grid relative to the goal, they might have found it expedient to use the 'direction' response, especially since using the 'state' response might have required them to move their cursors across the screen to click on the image of the goal instead. This behaviour is apparent in the models as well, where agents occasionally use a 'direction' response to approach the goal. Of course, some of this behaviour could also be attributed to attentional lapses (e.g., not noticing that the goal object was amongst the adjacent objects). Additionally, a select few participants appeared to have a strong preference for using only the arrows (or might have simply misunderstood the task), as evidenced by the few participants who exclusively used directions throughout the whole task (Fig. 2B).

Page 13 / 14: Typo - "in favour for the null hypothesis"

Thank you for spotting the typo - this has been **fixed**.

Figure 4: The caption for panels B and D are reversed

Fixed.

Pages 20 / 23: Typo - 'lik3ly'

Fixed.

Discussion: Typo - "people rely predominantly on vector-based strategies to heading in the general goal direction"

Fixed.

Reviewer #2 (William de Cothi): This is a thorough, elegant and well-written study investing goal-directed navigation strategies in humans and comparing them to reinforcement learning agents implementing meta learning via the recurrent connectivity in an LSTM layer (e.g. once learnt, network weights are fixed). The key benefit to this type of comparison is that the network can learn to approximate optimal behaviour on complex tasks, enabling the authors to probe both the optimality of human behaviour and the mechanisms behind it. In sum, I find this study uses sound and novel methods to provide useful insights into the mechanisms driving human goal-directed behaviour, and the preregistration of their experiments is to be applauded.

We thank the reviewer for the positive appraisal of our paper.

I have some suggestions to the authors:

I appreciate the authors attempt to dissociate direction- and state-based strategies which is not trivial to achieve. However, in the real-world these two things are not so distinct. The deterministic nature of (state,action) -> next_state transitions in navigating physical space means you can just as easily characterise the same policy as a sequence of actions (directions) or the sequence of states they lead to (which the authors refer to transitions). Technically, these both describe the same sequence of transitions, just one is in a state-based format and the other in a direction based format, so I take issue with authors' use of the term 'transitions' for meaning 'state-based'. The format in which participants choose to implement that policy will likely vary depending on state quantity and saliency - remembering every state is difficult and high value states are more memorable, meaning participants may choose to encode parts of the policy through low-value states via directions, and parts of the policy through high-value states via the states themselves. I would suggest:

1. The author's use the term 'state-based' rather than 'transition-based'

We appreciate the reviewer's point and agree that our use of 'transitions' could be potentially ambiguous. In our original manuscript, we were trying to use:

> (i) one set of terms that concisely referred to <u>the response chosen</u> by the participant (<u>'directions'</u> if they respond using the arrows, and <u>'states'</u> if they respond using the images that correspond to the subsequent state)
> (ii) a separate set of terms that concisely referred to the underlying <u>computation performed</u> as they made this selection (<u>'vector-based'</u> if arrows, and <u>'transition-based'</u> if images)

We prefer not to use <u>'state-based'</u> for the latter as it would not allow us to clearly distinguish between (i) responses chosen and (ii) underlying computations. However, we agree that one could interpret 'transitions' as referring to transitions between actions (directions) or state-action pairs. **In our revised paper, the first few times we introduce the concept, we use the term 'state transitions' instead of just 'transitions'** to make clear we are referring specifically to state transitions (i.e., state -> next_state transitions): e.g. "*associative, 'state transition-based' strategies allow us…*" (lines 84-85). For conciseness, we stick to using just 'transitions' in the rest of the paper, but we **clarify early on that we are referring specifically to state transitions**: "*Note that our use of 'transitions' in this paper refers specifically to computations involving transitions between states and not actions or directions.*" (lines 148-150).

2. The authors include something in their discussion on how these policies may not be so distinct in reality - I was actually expecting this to come in the paragraph starting line 712 so I would suggest it goes there

Yes, agreed. **In our revised paper, we have added this point to the paragraph in question**: "*Moreover, while we artificially dissociate between vector and state transition-based strategies, these strategies are likely not so distinct in real-world navigation, where we likely integrate information across both spatial and associative structures.*" (line 779-782)

3. In this part of discussion the authors also discuss potential goal-directed strategies not considered in their study/analysis - for example how does this fit with 'beaconing' and 'dead-reckoning' strategies?

In our revised paper, we have added a discussion on beaconing to the paragraph in question: "*At the same time, participants in our task could only observe states that they were adjacent to, while real-life navigation often involves using distal states as reference points [64–66] or, when the goal is visible, targets (i.e., 'beaconing') [67].*" (lines 776-779).

We believe our task captures some features of 'dead reckoning': in our participants first travel to a landmark before using vector-based computations to head to the goal, presumably keeping track of their position based on the direction and distance traveled from the landmark. **In our revised paper, we have added a reference to this in an earlier paragraph:** "*The crucial role of 'landmarks' in our task is in line with previous work suggesting an important role for landmarks in localisation [7,53,54], particularly during path integration or 'dead reckoning' [55,56].*" (lines 728-731).

New References:

56. Etienne AS, Maurer R, Séguinot V. Path integration in mammals and its interaction with visual landmarks. J Exp Biol. 1996;199: 201–209. doi:10.1242/jeb.199.1.201

64. Pfeiffer BE, Foster DJ. Hippocampal place-cell sequences depict future paths to remembered goals. Nature. 2013;497: 74–79. doi:10.1038/nature12112

65. Shapiro ML, Tanila H, Eichenbaum H. Cues that hippocampal place cells encode: Dynamic and hierarchical representation of local and distal stimuli. Hippocampus. 1997;7: 624–642. doi:10.1002/(SICI)1098-1063(1997)7:6<624::AID-HIPO5>3.0.CO;2-E

66. Knierim JJ, Hamilton DA. Framing Spatial Cognition: Neural Representations of Proximal and Distal Frames of Reference and Their Roles in Navigation. Physiol Rev. 2011;91: 1245–1279. doi:10.1152/physrev.00021.2010

67. O'Keefe J, Nadel L. The Hippocampus as a Cognitive Map. Oxford University Press: Oxford, UK. (1978). Oxford, UK: Oxford University Press; 1978. Available: http://www.cognitivemap.net/

In the analysis of LSTM units, 'spatial' cells are stated as being classified depending on their modulation in activity over quadrants of the environment. Whilst this would potentially fit a 'hippocampal place cell' view of spatial cells (although I'm not sure it would if the place cell was centred on the intersection of all quadrants?), my understanding is it would exclude an entorhinal grid cell as being spatial (e.g. a cell with spatially stable firing fields distributed across the environments)? Indeed, its hypothesised grid cells in entorhinal cortex play an important role in the neural computation of vectors (Bush et al., 2015; Banino et al., 2018). I would suggest:

4. If the above is correct, the authors use spatial stability within an environment as a criteria for categorising spatial cells e.g. correlation between activation rate maps constructed from, for e.g., 1st half vs 2nd half of trials, or alternating trials, or for alternating time steps.

Great suggestion. We used activity over quadrants for our cell classification because it allowed us to easily identify localised spatial firing patterns, while also determining how these patterns were affected when landmarks were placed in each of the four quadrants in the different environments. However, we agree with the reviewer's concerns that this results in a narrow definition of 'spatial' cells that mostly includes only cells with contiguous firing fields that fall primarily within one of the four quadrants.

To address these concerns, we have now repeated the analysis from Figure 4A using an alternative criterion - the linear correlation between spatial activation maps on even timesteps with that of odd timesteps either within the same environment, or in a different environment (with different landmark positions). As a control, we also compute the correlation between spatial activation maps on even timesteps for each unit with odd trials of all other units, similar to methods for classifying place cells based on stability in electrophysiological studies [1]. We classify cells as 'spatial' if their within-environment, within-cell correlation coefficients were within the 95th percentile of all shuffled within-environment correlation coefficients. Additionally, we classify cells as stable if their cross-environment correlation coefficients were within the 95th percentile of shuffled cross-environment correlation coefficients. The results are shown below:

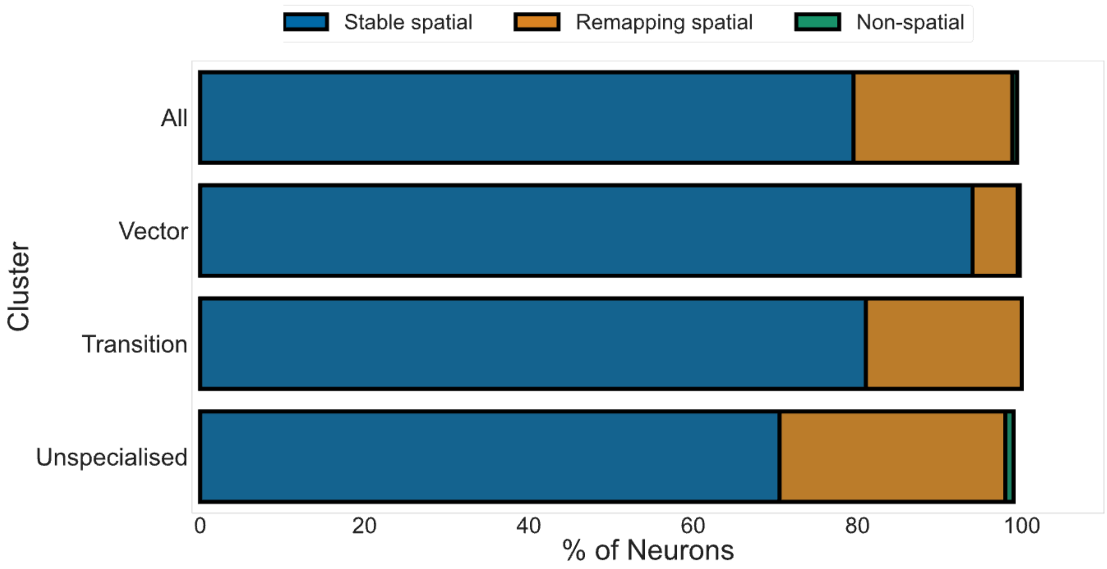

***Figure R3:** Stacked bar plots showing the proportion of response pattern types ('stable spatial', 'remapping spatial', or 'non-spatial') across all units or in the 'vector', 'transition', or 'unspecialised' clusters. 'Vector' units are more likely to have stable spatial responses, while 'transition' units are more likely to have remapping spatial responses.*

Using this metric, a vast majority of cells (79.50%) were classified as having 'stable spatial' response patterns, with some exhibiting 'remapping spatial' response patterns (19.35%) and a small minority having non-spatial response patterns. The proportion of 'stable spatial' response patterns identified using this metric is substantially larger than our quadrant-based metric. As the reviewer points out, such a metric imposes fewer constraints on the nature of these response patterns: for example, peak responses need not be confined within any specific quadrant, or in fact be contiguous at all (e.g., grid cells). However, when we looked at the relationship between spatial response patterns and the functional classification across units, we found a similar significant association between response pattern and functional classification (χ^2 = 40.38, d.f. = 4, p < .0001, n = 600). 'Vector' units were significantly less likely to have a 'remapping' spatial response (Adjusted Pearson residual = -4.02, p < .0001). This is in line with the idea that 'vector' units exploit generalisable spatial information across environments, while 'transition' units might be more likely to remap across different environmental contexts.

While an important sanity check, this classification criterion does not allow us to analyse the effect of landmark placement on activation patterns and how they may interact with space (e.g., forming 'conjunctive' responses). **In our revised paper,** we have left our original classification criteria intact but have **added this figure and a description of this analysis in the Supplementary Figures (Fig. S9)**. We also **refer to this additional analysis in the main text:** "*An alternative method for classifying activation patterns is by examining their stability across time points within and across environments. We observe similar results when classifying cells in this way: 'vector' units are more likely to have spatial patterns that are stable across environments, while 'transition'*

units are more likely to have spatial patterns that remap across different environments (Fig. S9)" (lines 504-508).

Some more minor points:

5. I agree with the the authors that one of the reasons to compare with deep RL agents is to form a comparison to a near-optimal strategy - therefore it would be helpful for them to should show that the networks' learning has plateaued during training, and is indeed near-optimal

We agree that this would be important to show. **In our revised paper, we have added the training curves to Supp Fig 4 and referred to it in the main text:** "*The performance of the RL agents improved throughout training and had plateaued by the end of the training run (Fig. S7)*" (lines 362-364).

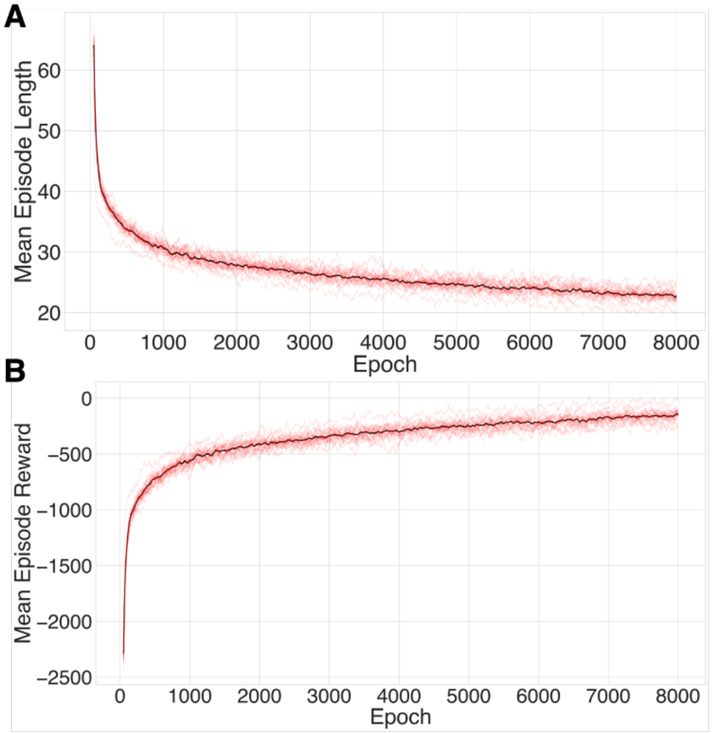

Figure R4: *Training curves for deep meta-RL agents showing how (A) mean episode length and (B) reward evolves with training epoch. Each red line represents the training curve for a single model with a single random seed, while the black line represents the mean across all models.*

6. Line 457 - the cells they describe here also resemble boundary cells found in mammalian subiculum (e.g. Lever et al., 2009)

Thank you for bringing this to our attention - **we have added a reference to these findings** ("*resembling boundary cells… and subiculum*" - line 492).

New Reference:

47. Lever C, Burton S, Jeewajee A, O'Keefe J, Burgess N. Boundary vector cells in the subiculum of the hippocampal formation. J Neurosci Off J Soc Neurosci. 2009;29: 9771–9777. doi:10.1523/JNEUROSCI.1319-09.2009

Reviewer #3: This study investigates the arbitration between direction-based choice behavior and transition-based choice behavior in the context of spatial navigation in open vs cluttered environments. Human behavior is analyzed and compared to the action policies of trained RL agents. Core conclusions include 1. that humans do better when endowed with the possibility to use both behavioral strategies, 2. humans tend to use a transition-based strategy close to landmarks/goals. Below is elaborated several conceptual queries aimed at helping to deepen our understanding of these results and relate them to the literature.

We thank the reviewer for their interesting questions and have performed a host of additional analyses to address them. We believe these additional analyses have substantially improved our understanding of human and model behaviour on our task.

One possible alternative to using landmarks is using the wall or boundaries. There is not any quantification of this behavior. For example, in experiment 2 do people use the wall more if there are fewer landmarks? Could this explain the flat curve for number of landmarks presented vs number of steps taken to goal?

This is indeed an interesting suggestion. **To address this question, we present new analyses looking at the role of boundaries of the environment.** Indeed, people's navigation accuracy improves after encountering the edge of the grid (Fig R5A; logistic mixed effects model - edge encountered: $\beta = 0.34$, SE = 0.068, $z = 5.12$, $p < .0001$). This effect is ever so slightly stronger when there are fewer landmarks (edge encountered x number of landmarks interaction: $\beta = -0.015$, SE = 0.0071, $z = -2.16$, $p = .03$). However, there does not seem to be any large variation in the amount of time participants spend on the edge across the landmark conditions (Fig R5B). Hence, we might conclude that participants' reliance on edges cannot fully account for the lack of effect of the number of landmarks, which is likely more a function of participants' memory constraints (see response to Reviewer 1). **In our revised paper, we have added these new figures into the supplementary (Fig. S5E/F)** for readers who might be curious about the role of edges.

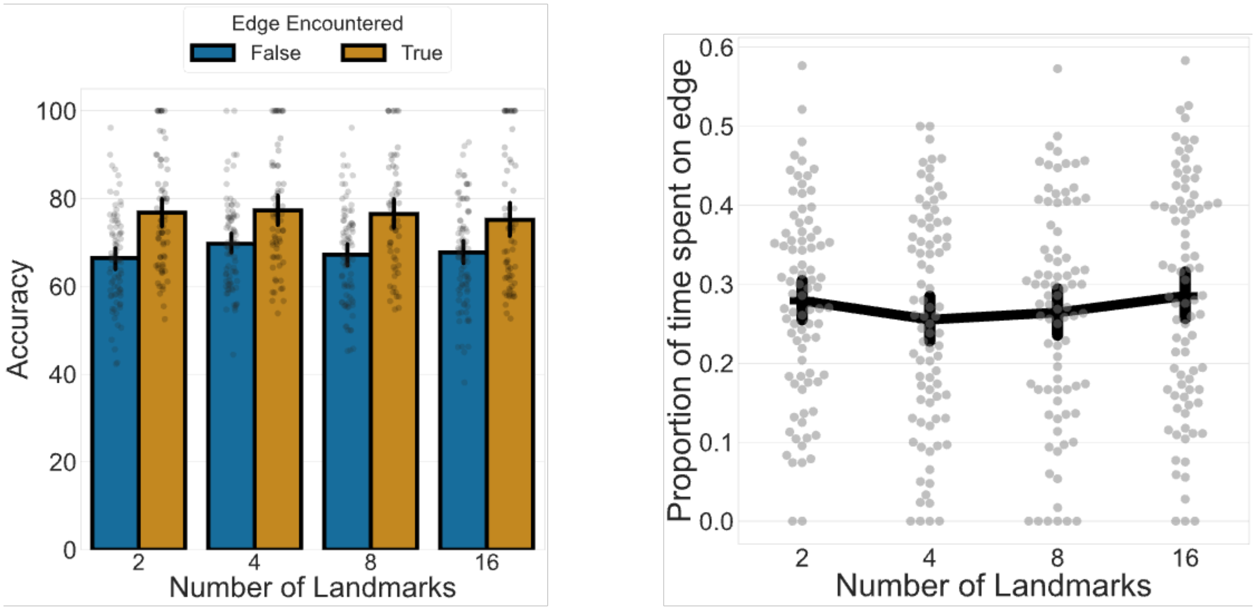

__Figure R5:__ left: Accuracy in navigation (y-axis) across the number of landmark conditions (x-axis) before and after the edge of the grid is encountered (hue). Each dot represents an individual participant and error bars represent the 95% CI. right: Proportion of time spent on each trial spent on the edge of the grid (y-axis) across the number of landmark conditions (x-axis). Each dot represents an individual participant and error bars represent the 95% CI.

Experiment 2: What was the average number of landmarks used in a single trial? If it is around 2 then maybe 2 landmarks is all you really need and this can explain the data. I saw percentages of directions vs state action choice but not a clear average number of different landmarks used.

To address this question, we performed a new analysis looking at the average number of landmarks used across trials with different numbers of landmarks. The mean number of landmarks that participants in Experiment 2 encountered on each trajectory is 2.21 (SD = 0.72), while the mean number of landmarks they actively clicked on (i.e., by using a state-based action) on each trajectory is 1.07 (SD = 0.65). This is in line with the reviewer's suggestion that participants only need to use relatively few landmarks to navigate effectively. Participants are more prone to using more landmarks on each trajectory when there are more available (Fig R6); however, even when there are 16 landmarks, participants only actively use 1.64 landmarks on average. **In our revised paper, we have added this figure into the Supplementary Figure examining the effect of the number of landmarks (Fig. S5D)**.

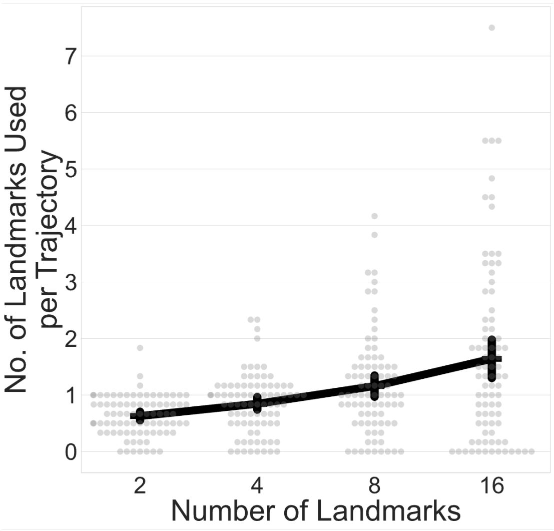

Figure R6: *Mean number of landmarks used (i.e., clicked on using a state-based response; y-axis) in the different number of landmark conditions. Each dot represents an individual participant and error bars represent the 95% CI.*

Experiment 2: The RL models use different amounts of noise to model different numbers of landmarks presented. If memory is a possible explanation for the same performance across different number of landmarks presented, are the landmarks presented last more likely to be used?

Confirmation that memory plays a role in the same performance across the number of landmarks can perhaps be more directly obtained from the data from Experiment 3, where we occasionally probed participants' memories of the location of two randomly selected landmarks and the goal after the navigation phase. As mentioned in our response to Reviewer 1 and can be seen in Fig. R1A, participants are more accurate on the memory probe on trials when there are fewer landmarks.

However, the reviewer also raises the interesting suggestion that landmark use might be affected by the order of landmark presentation. **To address this question, we performed new analyses looking at the relationship between landmark use and the order in which landmarks were presented.** We looked across all landmarks used (i.e., where participants had clicked on the image of the landmark) and calculated the proportion of landmarks which had been presented at each serial position in the map-reading phase (Fig R7). There appeared to be an effect of presentation order in the conditions with more landmarks (8 or 16). Specifically, while there was no strong recency effect as suggested by the reviewer, there appeared to be a *primacy* effect, where participants appeared more likely to use landmarks that appeared early on in the map-reading phase. We conducted an ANOVA by averaging the proportions for the first 2 ('early'), last 2 ('late'), and middle landmarks and found a significant main effect of order of first landmark presentation on landmark use in the 8 landmark and 16 landmark conditions (8 landmark: $F(2, 148) = 8.70$, $p < .001$; 16 landmark: $F(2, 140) = 9.48$, $p < .001$), while post-hoc t-tests revealed significant differences between the 'early' vs 'middle' and 'early' vs 'late' contrasts, but not the 'middle' vs 'late' contrast in both conditions. The existence of a primacy effect supports the idea

that landmark use depends on long-term (possibly hippocampal-dependent) memory of landmark locations. Moreover, the fact that only the first ~2 landmarks are over-represented in participants' landmark use sheds further light on how memory constraints might limit any potential benefits of additional landmarks. **In our revised paper, we have added this figure into a new Supplementary Figure on the patterns of landmark use (Fig. S6)**.

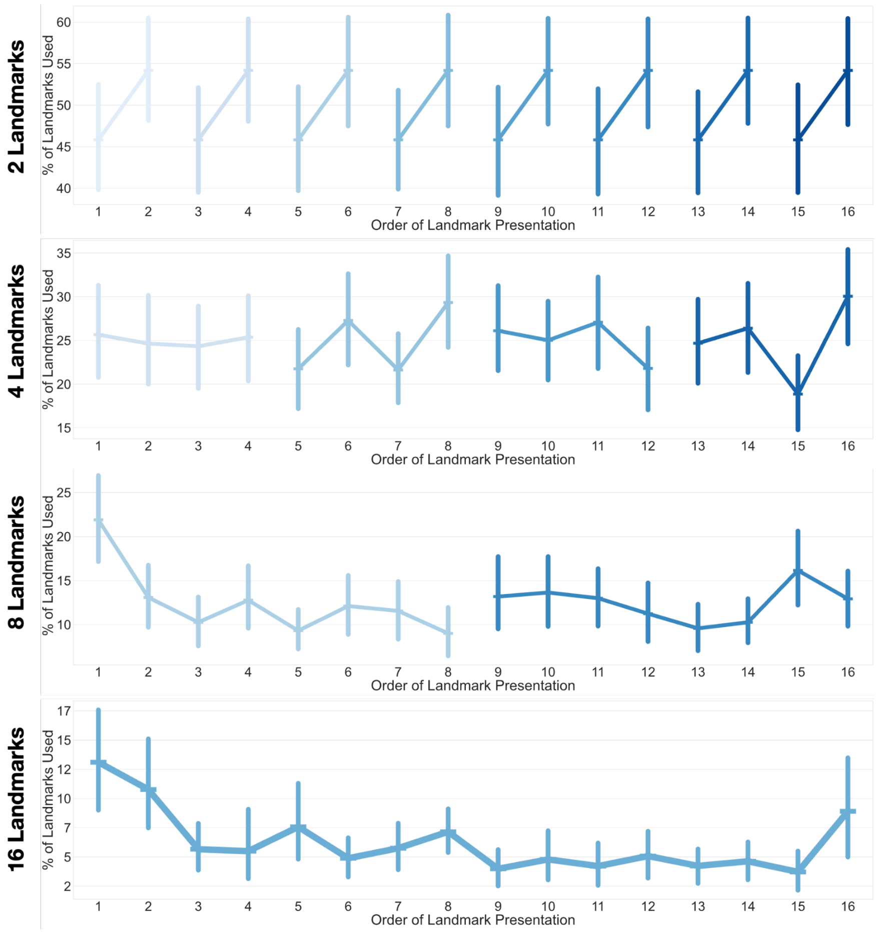

Figure R7: *Percentage of all landmarks used in the navigation phase (y-axis) that were presented in each serial position in the map-reading phase. Each plot represents a condition with a different number of landmarks. When there were more than 16 landmarks, the same landmarks were presented several times: the lightest hue in each plot indicates the first time the landmarks were presented, with progressively darker hues indicating each subsequent presentation. Error bars represent the 95% CI.*

Related to this, how does the agent behavior change if you make the goal/landmark encoding non-noisy? What are the predictions for this under the hypothesis that noisily encoding the landmarks/goals corresponds to a memory constraint.

Thank you for this interesting question. **To address this question, we performed new analyses where we re-trained models with non-noisy landmark encodings and examined how this affected model behaviour.**

As mentioned previously, we believe that memory constraints could account for why we do not see differences in performance when there are different numbers of landmarks. As seen in Figure R8A (and as an answer to the reviewer's later question), the deep RL agents with noisy landmark encodings perform similarly no matter how many landmarks there are, just like in humans. However, with non-noisy landmark encodings, we would expect that agents would actually perform better when there are more landmarks, as they would no longer be constrained by their memory of individual landmarks and would benefit from the increased landmark coverage of the environment. Indeed, when we re-trained and re-tested the agents with non-noisy landmark encodings, we observed that performance improves with increasing number of landmarks (Fig. R8B).

Moreover, the main purpose of using a 'state-based' response in our task is for agents to localise themselves in the environment, which in turn allows them to use 'direction-based' responses more effectively. When memory of landmarks is perfect, we might expect that the need for localisation might decrease, and agents would consequently use 'state-based' responses less. This turns out to be true (Fig. R8C vs R8D). Importantly, with noisy landmark encodings, agents might occasionally integrate over multiple landmarks to effectively localize themselves, especially in the high-landmark conditions where the location of each individual landmark is remembered less well. Hence, agents with non-noisy landmark encodings are prone to use multiple landmarks per trajectory, especially when there are more landmarks (Fig. R8E, as humans do: Fig. R6). However, agents with perfect landmark encodings end up using markedly fewer landmarks on each trajectory–using on average, less than one landmark on each run (Fig. R8F). **In our revised paper, we have included a description of these new simulations and results in the Supplementary Figures (Fig. S8).**

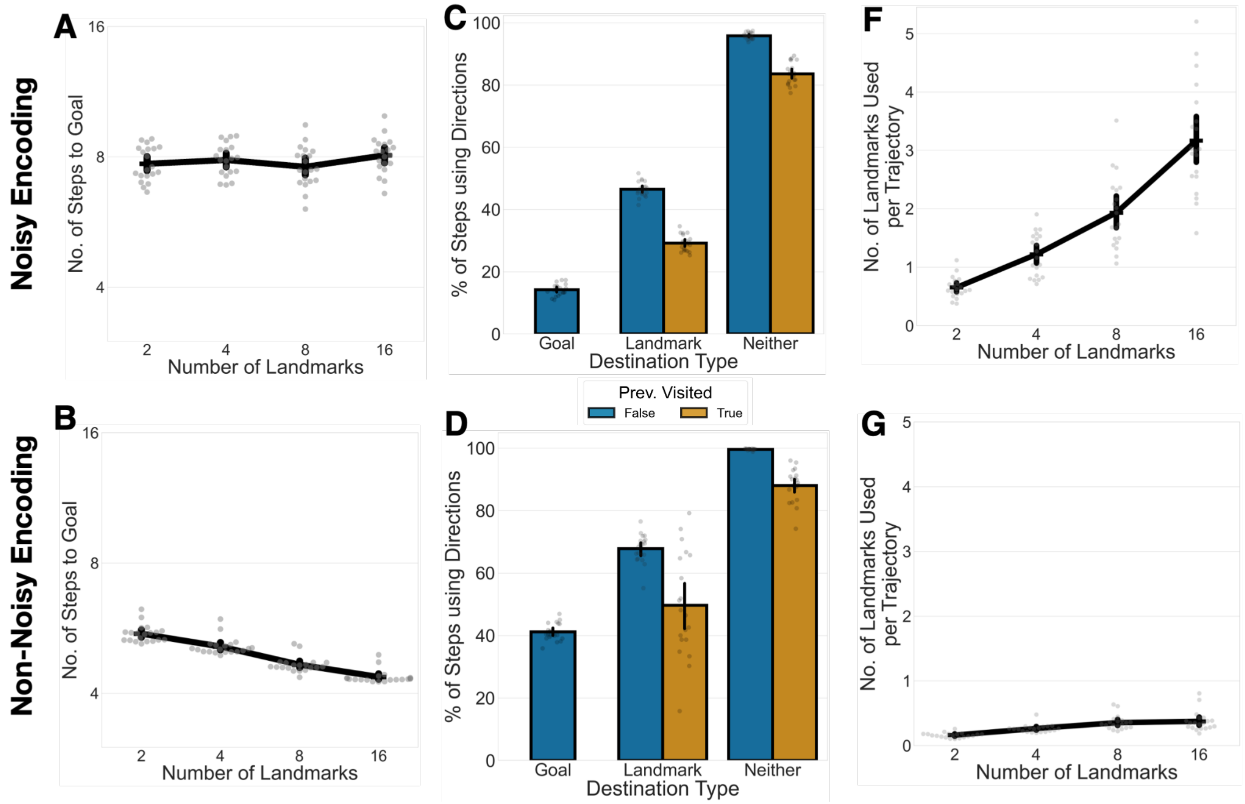

Figure R8: A: Performance as measured by number of steps taken to goal (y-axis; presented on a logarithmic scale) as a function of number of landmarks (x-axis) for models with noisy landmark and goal encodings. Each dot represents a model with a different initialisation seed, and the error bars represent the 95% CI. B: Same as A but for models with perfect memory of landmarks and goals. C: Models' use of vector-based responses (y-axis) as a function of destination type (i.e., goal, landmark, or non-landmark; x-axis) and whether the state had been visited before (colour of bar). Each dot represents the behaviour of an individual model, with error bars representing the 95% CI. D: Same as C but for models with perfect memory of landmarks and goals. F: Mean number of landmarks used (i.e., clicked on using a state-based response; y-axis) in the different number of landmark conditions. Each dot represents an individual model and error bars represent the 95% CI. G: Same as C but for models with perfect memory of landmarks and goals.

me

Experiment 2: Is there a pattern to which landmarks get used? Are landmarks closer to the goal more likely to get used? Are the landmarks that are first encountered more likely to be used (in order to reduce the uncertainty due to unknown starting location)?

We thank the reviewer for these interesting questions. **To address these, we have performed new analyses on the pattern of landmark use across time and space.** With regards to the latter question, the reviewer's intuition is right in that participants are most likely to use the first landmark they encounter on their trajectories to minimise their uncertainty of their current location as soon as they can. As can be seen in Figure R9A, when participants are adjacent to a landmark, they are most likely to use it (defined as clicking on the picture of the landmark) when it is the first time they encounter an adjacent landmark. With regards to the former question: ostensibly, participants are more likely to use landmarks when they are 4 steps away from the goal compared

to when they are closer or further away from the goal (Fig. R9B). However, this is likely an artifact of participants starting 4 steps away from the goal, combined with the fact that they are most likely to use the first landmark they encounter. When we plot landmark use separately for the first landmark and the subsequent landmarks that participants encountered (Fig. R9C and D), we observe a slight trend towards higher landmark use closer to the goal (with the exception of when participants are adjacent to the goal, when it is more sensible to click on the goal instead).

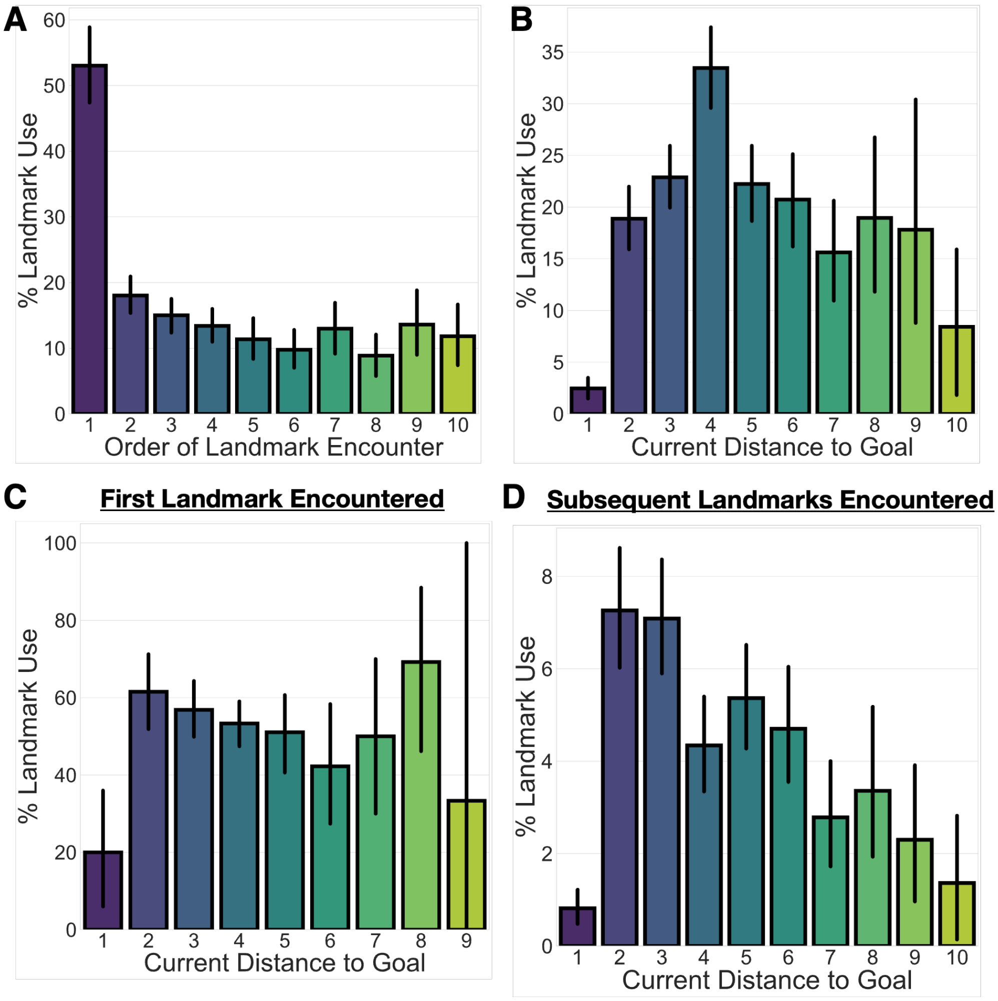

Figure R9: *A: Percentage of steps where participants used a landmark (defined as clicking on the image of a landmark on the state-based display; y-axis) as a function of the order in which they passed by the landmark on a navigation trial. Error bars represent the 95% CI. B: Percentage of steps where participants used a landmark (defined as clicking on the image of a landmark on the state-based display; y-axis) as a function of the order in which they passed the landmark on a navigation trial. Error bars represent the 95% CI. C: Same as B but focusing only on the first landmark participants pass by. D: Same as B and C but focusing only on subsequent landmarks that participants pass by.*

When we run a logistic mixed effects model predicting landmark use (on timepoints when participants are adjacent to a landmark but not adjacent to a goal), we found that participants were significantly more likely to use landmarks when this was the first landmark they

encountered (β = 2.60, SE = 0.26, z = 9.95, p < .0001) and slightly less likely to use landmarks when they were further away from the goal (β = - 0.06, SE = 0.030, z = -1.97, p = .049). There was also an interaction between the two factors, suggesting that the effect of goal distance was stronger when the landmark was the first landmark they encountered (β = -0.12, SE = 0.059, z = -2.03, p = .042). **In our revised paper, we include these figures and analyses in the new Supplementary Figure on the pattern of landmark use (Fig. S6).**

RL agent experiment: The RL agents were run on different numbers of presented landmarks. What does the plot recreating Figure 2E of number of steps to goal vs number of landmarks presented look like?

We have answered this above in our answer to the reviewer's question on agents with non-noisy landmark encodings in Figure R8A: like humans, agents (with noisy landmark encodings) perform similarly no matter how many number of landmarks there were. This is yet another point of similarity between the behaviour of humans and artificial agents. **In our revised paper, we now mention this in the main text:** "*Additionally, like humans, RL agents were also unaffected by the number of landmarks (Fig. S8A).*" (lines 416-417).

In general, its not clear why do the artificial agents develop transition vs state-based behavioral arbitration strategies? What ablations can be done to pinpoint the source of this? Its not clear why the artificial agents would care at all about transition vs state-based encoding. I find it a bit unconvincing to say that these agents are "approximately optimal" without a deeper interrogation into why they exhibit the pattern of action selection that they do.

Agents' behavioural policies for arbitrating between direction- and state-based responses likely resulted from the demands of our task. Specifically, because agents only know the location of a few 'landmark' states, neither strategy alone is sufficient for optimal performance. Relying solely on state-based responses is inefficient because agents do not know the full transition structure between states, and when encountering states for the first time, would have no knowledge of which state it should transition to. However, relying solely on direction-based responses is inefficient as it fails to exploit information about the few landmark states that agents do know. The importance of arbitrating these two strategies is evident in how agents perform when they are forced to use either only direction- or only state-based responses (Fig. 2A for humans, Fig. 3B for models). In these experiments and simulations, we have shown that performance suffers when humans and models were prohibited from freely choosing which response strategy to use, suggesting that the pattern of strategy selection observed in humans and models was crucial for optimal performance.

Direction- and state-based responses likely place different representational and computational demands on agents, leading them to develop different 'modules' for meeting these demands. Indeed, we have identified in our models units that appear to care more about either direction- or state-based responses (Fig. 4C), which we call 'vector' and 'state transition' modules. Lesioning

these modules lead to a double dissociation in the directions-only and states-only versions of the task (Fig. 4D/R10A), implying that the units in each module are indeed causally important for implementing the two different strategies. **To address the reviewer's question, we have also conducted new analyses examining how the behaviour of these lesioned models changed when they were allowed to freely arbitrate between response strategies.** We observe that lesioning the 'vector' and 'state transition' modules both lead to worse performance compared to lesioning unspecialised units and unlesioned models (Fig. R10A, $F(3, 57) = 86.45$, $p < .0001$). We then tried to identify the source of these performance deficits by examining the models' strategy use, and found that lesioning 'vector' modules led to models using direction-based responses less, while lesioning 'state transition' modules led to models relying more on state-based responses less (Fig. R10B, $F(3, 57) = 88.07$, $p < .0001$). In summary, lesions that shift the agents' biases towards using one strategy or another lead to a decrease in performance, supporting the idea that an optimal balance between the two strategies is crucial for performance.

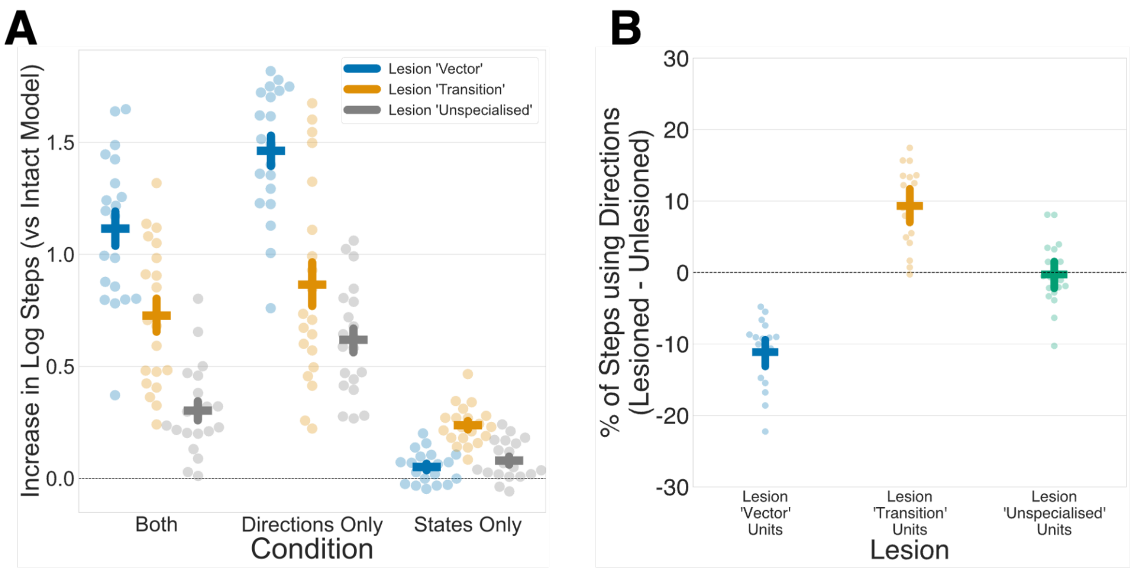

Figure R10: *A: Performance deficit of lesioned models (as measured by excess number of steps taken to get to the goal compared to an intact model) on the both, directions-only, and states-only conditions. Each dot represents one of the 20 trained models, and the line represents the mean, and the error bar represents the 95% confidence interval. B: Change in use of 'direction' actions after lesions to the 'vector', 'transition' and 'unspecialised' units, compared to the unlesioned models. Each dot represents one of the 20 trained models, and the line represents the mean, and the error bar represents the 95% confidence interval.*

We believe these additional analyses contribute significantly to our argument. **In our revised paper, we have added these results to Figure 4 and included these results in the main text:** "*Moreover, lesioning both 'vector' and 'transition' units lead to a greater performance deficit compared to lesioning 'unspecialised' units when agents could freely choose between either strategy ('vector' vs 'unspecialised': $t(19) = 12.76$, $p < .001$; 'transition' vs 'unspecialised': $t(19) = 5.23$, $p = .002$). However, these deficits likely resulted from different perturbations in agents' strategy use (Fig. 4E; $F(3, 57) = 88.07$, $p < .0001$). Lesioning 'vector' units pushed the agents away from using 'direction' responses ($t(19) = -7.61$, $p < .0001$), while lesioning*

‘transition’ units pushed agents away from using ‘state’ responses ($t(19) = 6.44$, $p < .0001$).” lines 534-541).

Figure 4 displays some unit responses in the trained network. It is striking that no grid cell-like responses are observed apparently. I think this would be important to discuss. For example, what are the differences in this agent architecture (vs e.g. Banino et al, Cueva/Wei) that lead to a lack of "grid cells". Have you tried applying spatial periodicity or "gridness" scoring to the unit responses?

To address this question, we performed additional analysis looking at the ‘gridness’ scores of unit responses. When we apply traditional measures for ‘gridness’ scoring (using code adapted from Banino et al. [2]), we do identify some units that ostensibly fit the criteria for ‘grid cells’. These cells have spatial autocorrelograms that exhibit 60 degree rotational symmetry within the 95th percentile null distribution (generated from field-shuffled activation maps). On the surface, these so-called ‘grid’ cells are more commonly found amongst the ‘vector’ units than the ‘transition’ and ‘unspecialised’ units ($\chi^2 = 7.20$, d.f. = 4, $p = .03$, $n = 600$), consistent with their suggested role in vector-based computations [2]. However, an inspection of the actual activation patterns of these supposed ‘grid cells’ suggests that these patterns are far from grid-like (Fig. R11). This is an established problem when applying traditional ‘gridness’ scoring methods, especially on responses from artificial neural networks: while ‘gridness’ scores focus on rotational symmetry and grid cells indeed have rotational symmetry, activation maps may also exhibit symmetry without necessarily being grid-like [3]. In any case, the gridness scores we get are markedly lower than those identified in animals and in other simulations involving neural networks.

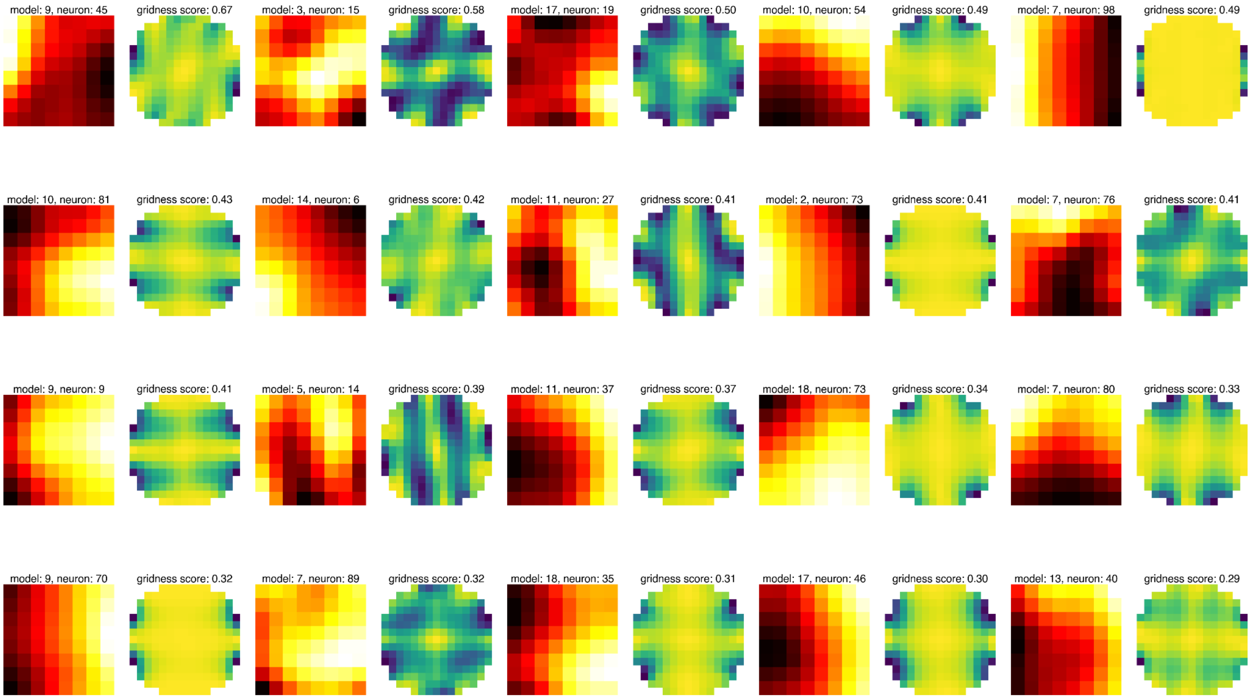

***Figure R11:** Sample of activation heatmaps (left) and spatial autocorrelograms (right) for units with the highest ‘gridness’ scores. ‘Gridness’ scores are shown on top of each unit’s spatial autocorrelogram.*

Importantly, our models have several important differences from previous studies that have purportedly identified grid cells in task-trained neural networks. In the first place, the networks in previous studies and in our study are trained on vastly different tasks. Most previous studies have trained recurrent networks on path integration tasks which attempt to mimic rodents freely roaming around an arena, often receiving (often two-dimensional) velocity vectors as inputs. In contrast, our networks were trained on a task where they were only allowed to move in the four cardinal directions around a square grid. There was no concept of velocity in the task (as networks always moved one square per timestep), and instead of velocity vectors, networks received as input information about landmarks.

Crucially, the networks in most previous studies were trained either with supervised objectives (e.g., to predict their current location) [2,4,5], or in more recent studies, with self-supervised objectives such as maintaining path invariance and separation between locations [6] or predictive coding [7]. In contrast, our networks were trained to maximise reward on our task (i.e., with a reinforcement learning objective). To our knowledge, no previous studies have identified grid cells in networks trained using only a reinforcement learning objective.

Moreover, in previous studies, the emergence of grid cells often depend on particular constraints and inductive biases built into the network, such as non-negativity [5] or particular target encodings on supervised tasks (e.g., Difference-of-Gaussian read-outs) [2,5]. These constraints appear critical to the emergence of grid cells, and networks trained on path-integration tasks without these constraints almost never develop grid cells [3], suggesting that a path-integration objective alone is not sufficient for the emergence of grid cells. We trained our networks without any of these constraints, and it is hence unsurprising that we do not observe grid cells.

Overall, the objective of our modelling exercise was not to identify grid cells, but to determine the types of representations that emerge when networks are trained, with minimal constraints, to solve a task that requires them to arbitrate between different navigation strategies to reach a goal. To this end, we are unsurprised at the lack of grid cells. Indeed, the lack of grid cells contributes to our growing understanding of the types of tasks and constraints that lead to the presence or absence of grid-like representations. Nevertheless, we agree that the absence of grid-like representations might be of particular interest to readers. **In our revised paper, we have added these analyses and discussion to the Appendix (Fig. S10) and referred to it in the main text:** "*Notably, we do not observe strong evidence for grid cells in our networks (see Fig. S10 for analysis and discussion).*" (lines 510-511).

A prominent theme in spatial cognition is the use of neural representations for structural generalization. For example, if two environments had roughly the same structure, then the same cell population might be activated while navigating in both environments. Is there any evidence of this in the RL agents?

Yes, we do see cells that appear to fire consistently in the same locations across different environments: for example, in Figure 4A of our paper, the response patterns of 'spatial' cells are

significantly modulated by spatial quadrant across all four environments we tested the agents in. These response patterns seem to be more common amongst the cells that contribute more towards the ‘direction’ actions, which is consistent with the reviewer’s suggestions that these cells might play a role in structural generalisation across environments. We have also used an alternative method to test the stability of firing patterns in our response to Reviewer 2 above. Again, we identified cells that appear to maintain similar firing patterns across two different environments, and found that these cells occur more frequently amongst cells that contribute more towards the ‘direction’ actions (Fig. R3). **In our revised paper, we have added a sentence when discussing these results emphasising the possible role of units with stable spatial activation patterns in structural generalisation:** *“The stability of spatial representations in ‘vector’ units hint at a role for these units in structural generalisation across spatial environments [26].”* (lines 508-510).

Presentation/minor comments:

What are the error bars in figure 2D? Please add info to caption.

Added - these are 95% confidence intervals.

Figure 1A and B. Might be clearer if the same map was used. You see the underlying structure that the participants dont see, then you see what the participants do see.

Thank you for this suggestion - we have **changed the figure** so that 1A and 1B reflect the same map. Additionally, we have edited Figure 1C so that the current and goal locations also correspond to locations on this same map.

Figure 4 labels are mixed up from caption.

Thank you for spotting this - this has been **fixed**.

Figure 5E caption: could be clearer that these are the principal components of the samples in the 8x8 environment

We have **amended the caption** to make this clearer.

Line 551 lik3ly

Fixed.

Line 670 - missing "work"

Fixed.

References in this Response:

1. Grijseels DM, Shaw K, Barry C, Hall CN. Choice of method of place cell classification determines the population of cells identified. PLOS Comput Biol. 2021;17: e1008835. doi:10.1371/journal.pcbi.1008835

2. Banino A, Barry C, Uria B, Blundell C, Lillicrap T, Mirowski P, et al. Vector-based navigation using grid-like representations in artificial agents. Nature. 2018;557: 429–433. doi:10.1038/s41586-018-0102-6

3. Schaeffer R, Khona M, Fiete IR. No Free Lunch from Deep Learning in Neuroscience: A Case Study through Models of the Entorhinal-Hippocampal Circuit. 2022. doi:10.1101/2022.08.07.503109

4. Cueva CJ, Wei X-X. Emergence of grid-like representations by training recurrent neural networks to perform spatial localization. arXiv; 2018. doi:10.48550/arXiv.1803.07770

5. Sorscher B, Mel GC, Ocko SA, Giocomo LM, Ganguli S. A unified theory for the computational and mechanistic origins of grid cells. Neuron. 2023;111: 121-137.e13. doi:10.1016/j.neuron.2022.10.003

6. Schaeffer R, Khona M, Ma T, Eyzaguirre C, Koyejo S, Fiete IR. Self-Supervised Learning of Representations for Space Generates Multi-Modular Grid Cells. 2023. Available: https://openreview.net/forum?id=8ox2vrQiTF

7. Tang M, Barron H, Bogacz R. Learning grid cells by predictive coding. arXiv; 2024. doi:10.48550/arXiv.2410.01022
