# Supplementary material for: Goal-directed navigation in humans and deep reinforcement learning agents relies on an adaptive mix of vector-based and transition-based strategies
Source: PLoS Biol. 2025 Jul 29;23(7):e3003296. doi: 10.1371/journal.pbio.3003296 (PMC12324678; doi:10.1371/journal.pbio.3003296)
Supplement: Attachment — Submitted filename: Response to Reviewers R2.pdf [file pbio.3003296.s017.pdf]

Thank you for the opportunity to revise our manuscript. In our response below, reviewers' comments are reproduced in black and our response is presented in blue. Changes to our manuscript are highlighted in **bold**.

Reviewer #3: I thank the authors for this thoughtful revision. Overall, the experimental design is clean, and the range of behavioral and model analyses is impressive. The manuscript clearly represents a significant amount of work and will be of interest to many researchers studying navigation, decision-making, and artificial agents.

We thank the reviewer for their positive comments on our manuscript.

That said, I still find myself seeking greater theoretical clarity around some of the core claims, particularly regarding the normative principles underlying arbitration between strategies. While the authors state that "agents' behavioural policies for arbitrating between direction- and state-based responses likely resulted from the demands of our task," and that these responses "place different representational and computational demands," the explanation remains somewhat opaque. Without a stronger normative or computational framing, it's difficult to evaluate what "optimal" arbitration actually means here—or whether the performance of DRL agents can be considered optimal in a meaningful sense. Potentially, the authors could soften the language surrounding "optimality" of arbitration and/or contextualize more specifically with respect to DRL agents (which may or may not be ``optimal").

We agree with the reviewer's point that our analyses do not allow strong conclusions about what arbitration strategies are 'optimal' for our task—at least not in the provable, mathematically guaranteed sense. Instead, the deep RL models allow us to approximate what a near-optimal (or at the very least, a sensible) strategy for solving our task might look like, subject to the assumptions built into the agents (e.g., that they attempt to maximise reward on the task, that they have noisy memory of landmarks, etc.) and certain assumptions about training (e.g., that they have converged near the global minima in their loss functions).

To address these points, we have **qualified our claims about optimality** in our manuscript: "*Assuming that agents have converged on near-optimal policies, this suggests that the strategies adopted by humans were also roughly optimal for this task, under the assumptions built into the deep RL agents.*" (lines 419-422). Where it is too cumbersome to caveat what we mean by "optimality", we have also replaced the word "optimal" with "effective" (e.g. line 382).

On the grid-like representation issue, I agree these are not grid-like units though some appear close to superpositions of plane waves. However, I'm unsure about the decision to largely set grid codes aside, especially since there is such a strong emphasis on the emergence of "representational geometry" from the "mammalian navigational system" in the DRL agents and the fact that, functionally, the authors link direction-based choice to vector-based navigation—a strategy closely tied to grid codes in the brain (e.g. Bush et al, Neuron 2015). Overall, the study is rigorous and thoughtful, however its focus on behavioral comparisons and the analysis of internal representations in artificial agents which do not exhibit key, and functionally relevant, spatial codes observed in biological organisms creates confusion for me regarding what the biological implications of this study are.

We agree that there is convincing evidence that grid cells are involved in implementing vector-based navigation strategies, and that the lack of grid-like representations in our networks despite the importance of vector-based strategies in our task is a notable omission. Unlike other modelling papers on grid-like representations, the purpose of our modelling exercise was not to identify grid codes (or, for that matter, any specific brain-like representations). Instead, we simply wanted to ask what types of representations *naturally* emerge from training networks on our task. Despite imposing minimal constraints on our networks, we did discover the emergence of units with a variety of brain-like firing patterns (place-like, landmark, conjunctive, border-like), each of which contributed differentially to the implementation of state transition- vs vector-based strategies. This suggests that these diverse representations arise due to the different representational demands of vector- vs transition-based strategies.

Of course, what is equally interesting is what representations *do not* naturally emerge. The absence of grid-like representations is indeed a glaring omission. However, the absence of grid-like representations is also consistent with previous work showing that training networks to path-integrate or use vector-based strategies alone is not sufficient for the emergence of grid codes (e.g., Schaeffer, Khona & Fiete, 2022). This hence raises an interesting question: why do brains use grid codes for vector-based navigation when vector-based navigation can be achieved without grid-like representations? Future work should more carefully examine the conditions that are sufficient for grid-like codes to emerge, which may be specific training objectives (e.g., supervised or predictive losses), biological constraints (e.g., non-negativity), or environments (e.g., continuous metric spaces vs grid-worlds).

To address these points, we have **edited the main text to emphasise the importance of grid cells in vector-based navigation:** "*Notably, despite the proposed role of grid cells in vector-based navigation [24,25], we do not observe strong evidence for grid cells in our networks"* (lines 505-507). We have also **extended the discussion after Figure S10** to highlight why the absence of grid-like representations in our networks might be notable and speculate what conditions are missing in our model that may have led to this absence.

The comments about training objectives and input structure are helpful, though, to be clear, other models (e.g., Sorscher et al, Tolman-Eichenbaum) have recovered grid-like codes without velocity input per se. The analogous strategy to Banino et al would be to pretrain supervisedly on position and then train using RL "on top" of the presumably resulting grid-like populations. Then, one could see if the DRL agents "naturally choose" to use these grid codes for direction-base navigation or not.

While modelling studies have discovered grid-like codes without explicit velocity input, they nevertheless share many important differences with our networks. In the first place, many previous modelling studies involve environments with continuous metric spaces (e.g. Sorscher et al.), while our model is trained on a grid-world environment. While the Tolman-Eichenbaum machine is trained in grid-like environments, it also has many notable differences from our networks. For example, TEM is trained with a predictive objective (vs. a reward-maximisation objective in our networks), has a vastly different network architecture (i.e., explicitly factorising structural and sensory codes and representing

their conjunction through Hebbian weights), and has other built-in biological constraints (e.g., non-negative firing). To address this point, **we have extended the discussion in Figure S10 to emphasise the differences between our model and TEM.**

We agree that it would be interesting to see if networks with pre-existing grid-like representations would take advantage of these representations for implementing vector-based strategies in our task. We leave this for future work, as studying the role of grid codes is not the primary motivation of the current work.

The section on structural generalization is promising but would benefit from more further details — specifically, what kinds of environments generalize well and where generalization breaks down. However, I wonder if there is sufficient systematic diversity on environment structure to examine this and I understand the authors may prefer to leave this aside as an issue not core to their overall goal in this study.

The question of when structural generalisation might be beneficial or detrimental is indeed a fascinating one. However, our task is limited to 8x8 2-D grid environments, which means that all environments share very similar underlying structures. As the reviewer suggests, this limited variability in environmental structure makes it hard for us to answer this question in a meaningful way.

In our task, the only source of variability in underlying structure arises in the 'cluttered' condition, where the layout of obstacles changes on every trial. We would hence expect that structural generalisation confers more benefits in the 'open field' environment, where all environments share the same underlying structure, than the 'cluttered' environment. Indeed, both humans and deep RL agents perform better in the 'open field' condition than in the 'cluttered' condition.

As the reviewer suggests, we have decided that a fuller exploration of this question is not central to the current study. Nevertheless, we agree that it would be interesting for future work to explore generalisation behaviour in a wider variety of environments, perhaps even those that do not obey Euclidean geometry. **We have added a sentence in the Discussion section to suggest this as an interesting direction for future study: "***For example, while meta-learning facilitates structural generalisation in similar environments, it would be interesting to see when and how generalisation breaks down in environments that lack the underlying structure seen during training."* (lines 743-746).
